# Evaluating the performance of verbal autopsy for assigning cause of death in older adults: A population‐based cohort study in Karonga, Malawi

**DOI:** 10.1111/tmi.14120

**Published:** 2025-05-13

**Authors:** Milly Marston, Alexandria Chung, Albert Dube, Estelle McLean, Samuel Clark, Amelia Crampin, Clara Calvert

**Affiliations:** ^1^ Department of Population Health London School of Hygiene and Tropical Medicine London UK; ^2^ Botswana Sexual Reproductive Health Initiative (BSRHI) Botswana Harvard Health Partnership, Princess Marina Hospital Gaborone Botswana; ^3^ Malawi Epidemiology and Intervention Research Unit Karonga Malawi; ^4^ Department of Sociology, Institute for Population Research, and Translational Data Analytics Institute The Ohio State University Columbus Ohio USA; ^5^ Usher Institute University of Edinburgh Edinburgh UK

**Keywords:** cause of death, East Africa, InsilicoVA, Malawi, mortality, verbal autopsy

## Abstract

**Background:**

Verbal autopsy, where a close caregiver or relative of someone who recently died reports on the signs, symptoms and circumstances preceding death, is useful for producing population‐based cause of death estimates. However, the performance of verbal autopsy for older adult deaths is poorly understood.

**Objectives:**

To evaluate the performance of verbal autopsy in assigning cause of death for adults aged 50+ in a rural area of Malawi.

**Methods:**

Cause of death was assigned to each death with a verbal autopsy in the Karonga Health and Demographic Surveillance site between 2002 and 2017 using two methods: (1) Physician review and (2) in silico verbal autopsy (a Bayesian probabilistic model). We calculated uncertainty in cause of death assignment for each method and calculated disagreement in cause of death between methods. Analyses were stratified by age group and sex.

**Results:**

A total of 2378 adult deaths were included (1360 aged 50+). Cause of death assignment showed greater uncertainty at older ages in both methods. For example, 59.7% of men aged 80+ were assigned a specific cause of death using physician review, versus 77.5% of men aged 30–49. Population‐level, broad cause of death distributions were similar across methods, but at the individual level there was over 30% disagreement on broad cause of death categories in those aged 50+.

**Conclusions:**

Verbal autopsy becomes more uncertain at assigning cause of death at older ages. The inclusion of any reports of medical diagnoses from a doctor and using a two‐stage process of cause of death assignment (with simple cause of deaths assigned using algorithms and more complex cases being reviewed by physicians) could improve cause of death ascertainment using verbal autopsy at older ages.

## INTRODUCTION

Dramatic improvements in life expectancy across many African countries mean that a much larger proportion of individuals are living to older ages [1], including a substantial proportion of people living with HIV who have been able to access ART treatment [2, 3]. This has been a public health success, but brings challenges for healthcare systems, shifting the burden of disease towards conditions common in older people, including increasing levels of multimorbidity. Tracking the changing burden of disease is important for informing healthcare planning to ensure adequate health system resourcing and the implementation of interventions to improve survival and quality of life. However, understanding the main causes of mortality is challenging in countries lacking civil registration and vital statistics (CRVS) systems.

Verbal autopsy (VA) is a tool used for estimating cause of death (CoD) in populations where most deaths occur outside health facilities, and has been used in many studies in Africa [4]. In a VA, a caregiver or relative of the recently deceased is asked about the signs, symptoms, and circumstances leading up to death [5]. Historically, these VAs were then reviewed by physicians to assign CoD but, due to cost and reproducibility concerns, there has been a movement towards instead using algorithms [6, 7]. It remains unclear, however, whether VA (and algorithms) are appropriate tools for assigning CoD in older ages where multimorbidity complicates CoD.

Studies reporting on CoD from VA in Africa, using either physicians or algorithms to assign CoD, indicate a higher proportion of older adult deaths are categorised as ‘unspecified or other’ (~10%–20%) compared to younger age groups [8, 9, 10, 11, 12, 13, 14]. Reasons for this may include reduced quality of questionnaire data, differences in disease presentation in older versus younger adults, and the higher burden of multimorbidity, which will not be captured when a single CoD is assigned, as is most often the case when assigning CoD from VA data. Health and demographic surveillance systems (HDSS) from Côte d'Ivoire, Burkina Faso, and South Africa reported higher proportions of incomplete VA data among adults 65+ compared to other adult age groups [11, 13, 15]; however, a Kenyan HDSS found less missing data in the over 65+ age group (10%) compared to those aged 15–49 (17%) [14].

Given the need for high‐quality CoD data in older adults in Africa and the increasing use of VA within routine surveillance acting as a starting point for CRVS systems [4, 16, 17], it is crucial to assess the use of VA for assigning CoD within older adults. We use population‐based data from the Karonga HDSS, operated by the Malawi Epidemiology Intervention and Research Unit, to evaluate the performance of VA in capturing CoD for adults aged 50+ in a rural area of Malawi. Comparing with younger adults, we assess levels of agreement between physicians in assigning CoD, look at the certainty with which one of the algorithms (InSilicoVA) assigns the most likely CoD, and compare the assigned CoD between physicians and InSilicoVA. Having identified causes where there is the largest disagreement in CoD assignment, we then randomly select VAs to be reviewed in detail by a clinical team member.

## METHODS

### Study setting and data collection

The Karonga HDSS was established in 2002, in the Karonga District of rural Northern Malawi, collecting information on all vital events (including births, deaths and migrations) in a population of over 30,000 individuals [18]. Key informants, who were members of the community, reported on vital events for a cluster of households. Following a reported death, a VA was conducted as soon as possible, after a 2‐week mourning period. An interviewer with clinical training visited the deceased's household and, if consent was given, filled in a semi‐structured VA form (locally developed tool, although similar to the World Health Organization standard VA questionnaire [19]). In the VA interview, a close relative or caregiver of the deceased was asked an open‐ended narrative question about the death, and then a series of closed questions about specific signs and symptoms. The patient‐held health record (Health Passport) was also examined and information recorded. The completed VA questionnaire was then reviewed by two physicians (physicians or clinical officers; hereafter referred to as physicians) who independently assigned an underlying CoD. Since 2012, due to the increasing complexity of CoD due to the emerging non‐communicable disease (NCD) burden (and, for example, people dying *with* HIV rather than *from* HIV), an ‘underlying’ and, if appropriate, a ‘direct’ and a ‘contributory’ cause were assigned. If the reviews were discordant from 2002 to 2005 a meeting was held where all the information was reviewed and a final CoD assigned. Since 2006, due to the increasing number of VAs requiring resolution, the discrepant VAs were referred to a third reviewer (always a physician) who could access both original reviews and then assigned the final CoD(s). Population‐based HIV surveys were conducted in this study population from 2007 to 2012, and information on HIV status from these surveys was available to the physicians. This analysis included VAs conducted in the Karonga HDSS from 2002 up to the end of 2017, with different physicians assigning CoD over the study period.

### Data preparation

Deaths to adults aged 15+ were retained for analysis if they had a final underlying CoD assigned by physicians. We grouped deaths by age at death (15–29, 30–49, 50–59, 60–79, 80+).

The categories for the CoD that were used by physicians are shown in Supplementary Table 1. In brief, the underlying CoD could be assigned to: (1) specific causes (e.g., ‘AIDS’, ‘Asthma’, ‘postpartum haemorrhage’); (2) more general causes, but within a subgroup of CoDs (e.g., ‘other/unspecified cardiovascular’) or (3) a much more general category (e.g., ‘other or unspecified non‐communicable disease’). We further grouped these causes into broad CoD groups (Supplementary Table 1) as follows: communicable disease, non‐communicable diseases, external cause of death, direct‐obstetric causes and indeterminate. This was done for both the CoD assigned by each individual physician and the final assigned CoD based on the consensus between physicians.

The signs and symptoms reported in the VA were prepared according to the input format required to run InSilicoVA, and were processed in the OpenVA package (version 1.1.1) using R (version 4.2.0) [20]. For each VA, each CoD was allocated a probability by InSilicoVA, with all CoDs adding to one for a given VA. For each death, we also assigned the ‘most likely’ CoD as the cause with the highest probability. If the highest probability was lower than 0.4 we categorised CoD as indeterminant. This is a somewhat arbitrary cut‐off but has been applied in many VA studies [21]. The CoD categories assigned by InSilicoVA are shown in Supplementary Table 2 and, as with physician review, these CoDs included more specific CoDs, where there were sufficient details in the VA, or more broad categories such as ‘other and unspecified non‐communicable disease’. The CoDs assigned from InSilicoVA were then grouped into broad categories in line with those used for the physician CoD.

Since the specific CoDs assigned in the physician review were not the same as those from InSilicoVA, we additionally created a variable harmonising the two, giving the most detailed breakdown possible (Supplementary Table 3).

### Data analysis

Analyses were conducted using Stata 18.0.

InsilicoVA calculates probabilities for underlying CoD; therefore, in our main analysis, we only consider the physician assignment of underlying CoD. For deaths from 2012 onwards, we calculated the percentage of deaths by age group and sex that also have direct and contributory CoDs assigned to them by physicians.

We first calculated the percentage of VAs where there was any disagreement between physicians, regardless of whether there were two or three physicians, by age group and sex for both broad categories and more specific categories of CoD. We also looked at how the percentage of deaths assigned as ‘Other and Unspecified’ varied by age group and sex.

We explored how InSilicoVA performs in older people compared to younger people. First, we looked at the probability of the most probable CoD, specifically looking at the distribution of probabilities by age group and sex. Subsequently, as with the physicians, we looked at the percentage of older people who were assigned to the ‘Other and unspecified group’ compared with other age groups, by sex.

Comparison of CoD from InSilicoVA and physicians was done in two ways. Firstly, we looked at how many physician CoDs matched the CoD assigned by InSilicoVA at the individual level, using the detailed harmonised CoD variable and the broader CoD variable, stratified by sex and age group. We then tabulated the physician CoD with InSilicoVA assigned most probable CoD for those who did not match, to see if there was any pattern to the disagreements. Secondly, we compared the population cause‐specific mortality fractions (i.e., the percentage of deaths assigned to each cause) from InSilicoVA and physician review.

We used chi‐square tests to assess for evidence of differences between age groups.

### Sensitivity analysis

We conducted a sensitivity analysis looking at how many physician CoDs matched the CoD assigned by InSilicoVA for the broader CoD category variable among VAs for deaths from 2012 onwards, considering there to be a match if any of the direct, underlying, or contributory causes assigned by the physician matched the cause group assigned by InSilicoVA. These results were stratified by sex and age group.

### Case studies

To understand the discrepancies between InsilicoVA and physician review CoD assignment, individual case studies were selected and reviewed by a clinical team member (Alex Chung). To yield the most useful information, cases were selected randomly from the disease category in which there was the largest disagreement in CoD assignment in adults 50+ between InSilicoVA and physician review. The signs and symptoms and narrative description of the circumstances leading up to death reported in the VA, the CoD assigned by InSilicoVA and by physicians (including direct and contributory cause of death, where available), any free text comments from physicians and, where available, the health passport photographs were reviewed and extracted into a short narrative.

#### Ethical approval

Ethical approval for the HDSS including VA data collection was granted by the National Health Sciences Committee of Malawi [#419 and #20/11/2641] ethics. Ethical approval for this analysis was granted by the London School of Hygiene and Tropical Medicine ethics committee.

## RESULTS

### Study population

There were 2429 deaths recorded between 2002 and 2017 to adults aged 15+, all of which had a CoD assigned by InSilicoVA, and 2378 (98.0%) had a final physician review and were therefore retained for this analysis. A total of 1360 (55.9%) were deaths to those aged 50+ (Table 1).

**TABLE 1:** Age and sex distribution of deaths in the study area with a final physician reviewed cause of death assigned.

Age group	Men		Women		Total	
	%	*n*	%	*n*	%	*n*
15–29	12.0%	142	13.4%	160	12.7%	302
30–49	35.0%	415	27.8%	332	31.4%	747
50–59	12.3%	146	9.4%	112	10.9%	258
60–79	24.7%	293	29.5%	352	27.1%	645
80+	16.0%	189	19.9%	237	17.9%	426
Total	100.0%	1185	100.0%	1193	100.0%	2378

Of the 897 VAs conducted since 2012 (which included direct and contributory CoDs), 60% had only an underlying CoD assigned by physicians, 20% had an underlying and direct CoD, 15% had an underlying and contributory CoD, and 5% had a direct, underlying, and contributory CoD. There was some variation by age and sex (Supplementary Figure 1).

### Physician review cause of death assignment

Disagreement between the first two physicians on broad CoD was slightly higher in men compared to women at all ages, and there were differences by age with the same pattern for men and women (Table 2). Disagreement was highest for the oldest age group (80+), 24.3% of cases for men and 21.0% for women; this compared, for example, to 15.9% and 12.7% for men and women, respectively, in the 30–49‐year‐olds (*p*‐value 0.15 for men and 0.07 for women). There were similar age and sex patterns using more specific CoDs, but with higher levels of disagreement (Table 2).

**TABLE 2:** Percentage of deaths where physicians disagree with the cause of death, by age group, sex, and broad or specific cause.

Age group	Broad cause			Specific cause		
	Men	Women	Total	Men	Women	Total
	(*n* = 1185)	(*n* = 1193)	(*n* = 2378)	(*n* = 1185)	(*n* = 1193)	(*n* = 2378)
15–29	19.4%	16.3%	17.7%	47.5%	38.8%	42.8%
30–49	15.9%	12.7%	14.5%	38.6%	34.1%	36.6%
50–59	17.1%	16.1%	16.7%	50.0%	41.1%	46.1%
60–79	19.9%	19.4%	19.7%	48.1%	45.1%	46.5%
80+	24.3%	21.0%	22.5%	53.4%	53.6%	53.6%
Total	18.8%	17.1%	18.0%	45.8%	42.5%	44.1%

*Note*: *p*‐values from Chi^2^ tests for each group are as follows: percentage not matched on broad cause‐ men *p*‐value = 0.16, women *p*‐value = 0.07, overall *p*‐value = 0.009; percentage not matched on specific cause‐ men *p*‐value = 0.005, women *p*‐value = <0.001, total *p*‐value = <0.001.

After excluding external CoDs, adults 60+ and adults aged 15–29 were more likely to be assigned to a more general category of ‘other or unspecified’ or no CoD assigned (*p* <0.005, Table 3). For those 80+, 16.6% of deaths for men and 20.7% for women had no CoD assigned either due to missing information on the questionnaire or a single CoD not being isolated.

**TABLE 3:** Percentage of deaths assigned a specific cause or given an ‘other and unspecified cause of death’ as the final cause of death by physicians, by age group and sex.

Age group	Men (*n* = 1048^a^)				Women (*n* = 1166^a^)			
	Specific cause assigned	Other and unspecified within a specific cause group assigned	Other and unspecified within a broad cause group assigned	No cause of death assigned	Specific cause assigned	Other and unspecified within a specific cause group assigned	Other and unspecified within a broad cause group assigned	No cause of death assigned
	(*n* = 739)	(*n* = 176)	(*n* = 33)	(*n* = 100)	(*n* = 870)	(*n* = 149)	(*n* = 39)	(*n* = 108)
15–29	62.5%	27.1%	4.2%	6.3%	71.3%	15.3%	6.4%	7.0%
30–49	77.5%	12.1%	1.6%	8.8%	84.8%	10.6%	1.6%	3.1%
50–59	76.9%	12.3%	3.1%	7.7%	80.0%	14.3%	1.9%	3.8%
60–79	68.1%	18.8%	5.1%	8.0%	72.9%	14.0%	3.1%	10.0%
80+	59.7%	21.0%	2.8%	16.6%	62.9%	11.6%	4.7%	20.7%
Total	70.5%	16.8%	3.2%	9.5%	74.6%	12.8%	3.3%	9.3%

^a^ External causes of death were excluded (men *n* = 137, women *n* = 27). *p*‐values from the Chi^2^ testing for evidence of differences between age groups: men *p*‐value<0.001; women *p*‐value<0.001.

### InsilicoVA cause of death assignment

The probability calculated for the top cause was lower among older adults compared with younger adults. For adults aged 80+, for example, half the deaths had a probability of 0.93 or lower, compared to a probability of 0.99 or lower for half the deaths for adults 15–49 years old (Supplementary Table 4). There was a decreasing percentage of deaths where the most probable CoD assigned by InsilicoVA had a probability over 0.90 with increasing age group (Supplementary Table 5).

There was also less likely to be a specific CoD identified for older people (*p* <0.005, Table 4). Up to age 60, most deaths were assigned a specific CoD, with between 6% and 17% assigned to the more general other and unspecified category. However, this increased from age 60, with 29.5% and 28.9% of deaths to those aged 60–79 and 80+, respectively, in the other and unspecified category for men. For women, this was much higher at 30.8% and 45.4%, respectively.

**TABLE 4:** Distribution of cause of death assigned by InSilicoVA, by age group and sex.

Age group	Men (*n* = 1056^a^)			Women (*n* = 1161^a^)		
	Specific cause assigned	Other and unspecified within a broad cause group assigned	No cause of death assigned (probability<0.4)	Specific cause assigned	Other and unspecified within a broad cause group assigned	No cause of death assigned (probability<0.4)
	(*n* = 812)	(*n* = 216)	(*n* = 28)	(*n* = 860)	(*n* = 272)	(*n* = 29)
15–29	79.8%	17.0%	3.2%	83.3%	15.4%	1.3%
30–49	83.4%	13.2%	3.4%	92.6%	5.9%	1.6%
50–59	82.1%	14.2%	3.7%	80.0%	17.1%	2.9%
60–79	68.0%	29.5%	2.6%	66.7%	30.8%	2.6%
80+	71.1%	28.9%	0.0%	50.2%	45.4%	4.4%
Total	76.7%	20.5%	2.7%	74.1%	23.4%	2.5%

^a^ Excluding external causes of death (men *n* = 129, women *n* = 32); men Chi^2^ *p*‐value<0.001, women Chi^2^ *p*‐value<0.001.

### Comparing physician review and InsilicoVA: Individual‐level cause of death

Using broad CoD, there was no difference in the level of disagreement between InSilicoVA and physician review by age for men. For women, the disagreement increased slightly as age increased (Table 5). Among over 50s, there was disagreement on the broad cause for 33.3% of deaths (*N =* 209) for men and *32.4*% (*N =* 227) for women. For 2012 onwards, we repeated the analysis with a non‐match being no match for underlying, direct and contributory cause. Overall, this reduced those not matching on broad cause from 26.5% to 21.4%. The largest reductions were for men 80+ with non‐matches reducing from 22.0% to 15.9% and women aged 50–59 from 18.5% to 13.2% (Supplementary Tables 6 and 7).

**TABLE 5:** Percentages of broad cause of death and specific harmonised cause of death disagreement between InSilicoVA and physician review by sex.

Age group	Percentage not matched on broad cause			Percentage not matched on specific cause		
	Men	Women	Total	Men	Women	Total
	(*n* = 1185)	(*n* = 1193)	(*n* = 2378)	(*n* = 1185)	(*n* = 1193)	(*n* = 2378)
15–29	22.5%	28.7%	26.2%	54.7%	58.9%	56.9%
30–49	32.5%	23.2%	28.6%	52.0%	44.5%	48.7%
50–59	33.6%	29.5%	32.6%	56.1%	50.4%	53.6%
60–79	34.5%	29.8%	32.2%	69.2%	60.1%	64.2%
80+	31.2%	41.3%	36.8%	68.1%	64.0%	65.8%
Total	31.7%	30.1%	31.2%	59.7%	55.5%	57.6%

*Note*: *P‐*values for Chi^2^ tests for evidence of a difference between age groups by sex are as follows: Percentage not matched on broad cause‐ men *p*‐value = 0.14, women *p*‐value = 0.002, overall *p*‐value = 0.033; Percentage not matched on specific cause‐ men *p*‐value<0.001, women *p*‐value<0.001, total *p*‐value = <0.001.

For those broad CoDs that did not match for those aged over 50, 56.9% for men and 56.4% for women was a mismatch between communicable and non‐communicable diseases (Table 6). Additionally, 18.2% of male deaths and 23.8% of female deaths were indeterminate by physician review but assigned as a non‐communicable disease by InSilicoVA. When also considering direct and contributory CoD, there was a slight reduction in the percentages of deaths to 50+ year olds with disagreement where there was a mismatch between communicable and non‐communicable diseases (Supplementary Tables 8 and 9).

**TABLE 6:** Percentages of non‐matching broad cause of death between InSilicoVA and physician review by sex (*N* = 209 for men and *N* = 227 for women).

InSilicoVA	Physician review				
	Communicable disease	Non‐communicable disease	External	Indeterminate	Total
	*n* (%)	*n* (%)	*n* (%)	*n* (%)	*n* (%)
Men					
Communicable Disease	‐	69 (33.0%)	5 (2.4%)	13 (6.2%)	87 (41.6%)
Non‐communicable disease	50 (23.9%)	‐	7 (3.4%)	38 (18.2%)	95 (45.5%)
External	1 (0.5%)	11 (5.3%)	‐	7 (3.4%)	19 (9.1%)
Indeterminate	0 (0.0%)	6 (2.9%)	2 (1.0%)	‐	8 (3.8%)
Total	51 (24.4%)	86 (41.2%)	14 (6.7%)	58 (27.8%)	209 (100%)
Women					
Communicable disease	‐	70 (30.8%)	2 (0.9%)	23 (10.1%)	95 (41.9%)
Non‐communicable disease	58 (25.6%)	‐	0 (0.0%)	54 (23.8%)	112 (49.3%)
External	1 (0.4%)	5 (2.2%)	‐	1 (0.4%)	7 (3.1%)
Indeterminate	5 (2.2%)	8 (3.5%)	0 (0.0%)	‐	13 (5.7%)
Total	64 (28.2%)	83 (36.6%)	2 (0.9%)	78 (34.4%)	227 (100%)

A more detailed breakdown of physician review CoD compared to InSilicoVA is provided in Supplementary Tables 10, 11. The biggest discrepancy between InSilicoVA and physician review was noted for digestive neoplasm, where the overall percentage of deaths attributed to this was 11.4% by InSilicoVA and 2.1% by physician review, with 133 deaths assigned to this by InsilicoVA but not physician review. For those assigned digestive neoplasm in InSilicoVA, the non‐harmonised physician review assigned 16.2% to unspecified/other gastrointestinal disorders, 11.3% to liver cirrhosis, and 10.0% to HIV/AIDS, with the rest distributed across other CoDs (Supplementary Table 12).

### Comparing physician review and InsilicoVA: Population‐level cause‐specific mortality fractions

Physician review was more likely to yield indeterminate CoD than InSilicoVA, and this increased in older age groups. Among women, for example, the percentage of deaths that were indeterminate based on physician review increased from 4.2% among deaths to 15–29year‐olds to 15.9% among 80+ year olds (Supplementary Table S1) whereas for InsilicoVA it remained below 3.5% across age groups.

**FIGURE 1:**
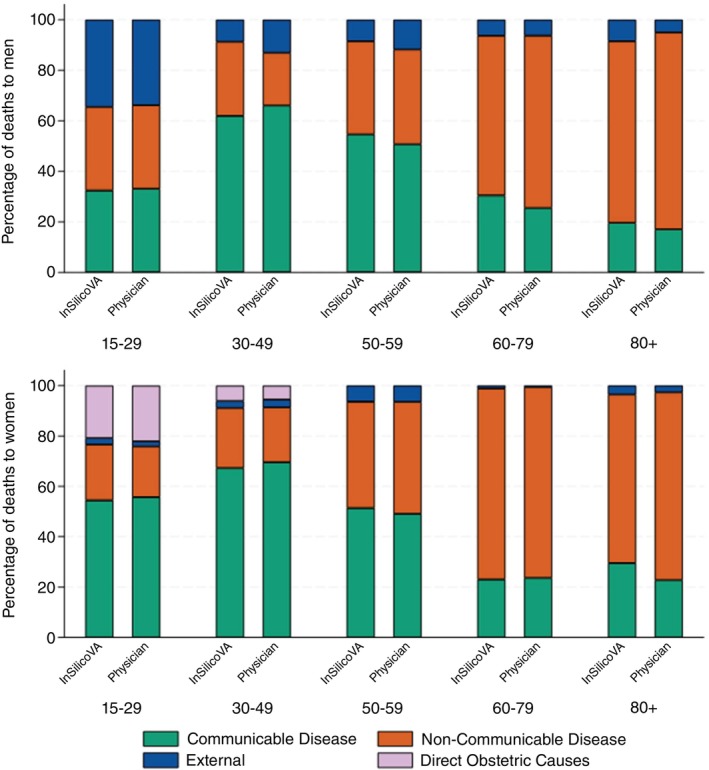
Distribution of broad cause of death by age group comparing the most likely cause of death from InSilicoVA and the underlying cause of death from physician review for (a) men and (b) women.

After excluding deaths assigned as indeterminate, the percentage of deaths attributed to broad causes was similar between InSilicoVA and physician review at a population level (Figure 1).

In 50–59‐year‐olds, the harmonised leading CoD was TB/HIV/AIDS for both types of VA assessment; however, the percentage attributed to this was much higher for men in the physician review at 39.1% compared to 27.3% with InSilicoVA. For those aged 60+, the leading CoD, unspecified cardiac disease or stroke, was the same between InSilicoVA and physician review. The second leading COD for InSilicoVA was digestive neoplasms for all age groups and both men and women, with the exception of women aged 80+. This cause did not come up as any of the five leading causes in the physician review; however, other and unspecified NCD was present in the second or third leading CoD by physicians for all groups (Supplementary Table 14).

### Case studies

As reported, there were particularly high levels of disagreement between the methods for digestive neoplasms; 142 deaths to adults 50+ were attributed to digestive neoplasm by InSilicoVA, of which only nine were given the same cause by physician review (Supplementary Table 10). Six VAs were randomly selected for review to ascertain why this discrepancy may have occurred. None of these VAs had health passport information, but all other sources were reviewed. The first two cases are shown in Supplementary Panel 1, and the details for the remaining four cases in Supplementary Panel 2.

Case 1 is a 56‐year‐old female where InSilicoVA has assigned digestive neoplasm with a high probability (*p* = 0.9998), but she was given a diagnosis of cervical/uterine neoplasm by physician review (with a consensus across three physicians). The clinical symptoms from the VA questionnaire suggest progression of an abdominal pathology but no specific information to directly suggest a cervical or uterine cancer. Information available in the narrative, however, gives a more detailed picture of the deceased's medical history including a description of her operation and the formation of a vesical‐vaginal‐colonic fistula, a complication which can arise from late‐stage gynaecological tumours. This extra information is nuanced and not captured in the closed questions which may account for the discrepancy in CoD assignment.

The symptoms provided by the questionnaire for Case 2 suggested a progressive gastrointestinal disease over the course of 3 months but did not seem to capture the potential diagnosis of tropical splenomegaly syndrome (also known as Hyperactive Malarial Splenomegaly) as reported in the narrative. Tropical splenomegaly syndrome is associated with an abnormal immune response to malarial infections and is not a form of cancer. All three physicians assigned the death as ‘Gastro intestinal disorder (unspec/other)’, with no mention of cancer in their notes.

While in the first two case studies there was no disagreement between the reviewing physicians for the underlying CoD, there was some disagreement between physicians in the other case studies (Supplementary Panel 2). In general, InSilicoVA appeared to give high certainty in diagnosing gastrointestinal malignancies in cases with non‐specific gastrointestinal symptoms (Cases 3 and 5; Supplementary Panel 2).

## DISCUSSION

Using VA data from nearly 2378 deaths in rural Malawi, we have shown both InSilicoVA and physician review become more uncertain in assigning CoD with increasing age. We found increased disagreement between physicians looking at the same death and reduced mean probabilities for the most likely CoD assigned by InSilicoVA with age. Both methods of CoD assignment were less likely to assign a specific CoD with increasing age. This is not surprising given that the prevalence of multimorbidity increases with older age [22, 23]. Our case studies, and as has been reported elsewhere [22, 23, 24], confirm an array of non‐specific symptoms which could correspond to many conditions and diseases in older people, contributing to the higher degree of uncertainty in the older age group compared to younger ages.

Physicians were more likely than the InSilicoVA algorithm to assign an unknown CoD, despite physicians having access to additional information, including the VA narrative description provided and health passports. This may be due to conceptual differences in CoD assignment between the two methods; while InSilicoVA identifies the most probable CoD and generally gives a non‐zero probability for all CoDs for each VA, physicians will only assign a specific CoD if they feel they can rule others out. Despite the known increase in multimorbidity in older people, the most probable CoD was still assigned a very high probability by InSilicoVA, whereas we might have expected it to be distributed more evenly over multiple CoDs.

Population‐level agreement on broad CoD categories between InSilicoVA and physician review was generally similar across all ages, consistent with cause‐specific mortality fractions in other African studies [25]. However, at the individual level, there was increasing disagreement between the methods with age. For example, there was disagreement between broad CoD for 37% of deaths to those aged 50+, rising to 66% when comparing more specific CoD. We found particularly large discrepancies for some specific CoD, notably digestive neoplasms, acute respiratory infections, and acute abdomen.

For digestive neoplasms, only nine of the 142 deaths of adults aged 50+ identified by InSilicoVA were also assigned to this by physicians, who tended to categorise these deaths under unspecified/other gastrointestinal disorder, HIV/TB, liver cirrhosis, and unspecified/other neoplasms. The International Agency for Research on Cancer reported the age‐standardised cancer mortality rates for adults aged 50+ in sub‐Saharan Africa to be highest for prostate and cervical cancer, with oesophagus and stomach cancer having much lower rates [26, 27]. InsilicoVA assigned 11.1% of deaths assigned to digestive neoplasms and only 2.9% to reproductive neoplasms, mirroring findings in South Africa [28] suggesting digestive neoplasms are being overestimated by InSilicoVA. This could be explained by the presentation of many late‐stage malignancies, where there is frequently intrabdominal spread with subsequent abdominal and gastrointestinal symptoms. Closed questions in the VA, used by InSilicoVA, are arguably insufficient for distinguishing specific CoDs in complex cases, including in older people Physicians assigning CoD in the Karonga HDSS draw on a wider range of data sources, including any diagnosis data available from health facilities as well as a narrative description of the circumstances leading up to death (often including the timing of symptoms relative to one another), which will lead to more accurate CoDs being assigned for complex cases.

The strengths of this paper include the use of the Karonga HDSS data, which provides high quality and comprehensive data on CoD. Unlike many other HDSS, Karonga has continued to use physician review and not rely only on algorithms. The HDSS also collects health records alongside the standard signs and symptoms, which allows a more detailed picture of a person's death to be formed, helping towards understanding why different CoDs might be diagnosed. Additionally, from 2012, the physician reviews have included direct and contributory CoD, enabling a more complex picture of CoD.

One of the challenges in this study is not having a true gold standard to compare the CoD assigned by physician review and algorithms to. While a full diagnostic autopsy would provide gold standard information on CoD, these are rarely conducted in Malawi. The reasons for this are not clear but evidence from other settings in Africa suggests this might be due to health system, cultural and social factors [29, 30]. There is some ongoing work to establish the acceptability and performance of minimally invasive tissue sample (MITS) to establish CoD in Malawi, but this is focussed on paediatric deaths [31, 32]. In the absence of autopsy data, physician review is typically considered the closest to a gold standard, since they also have access to information beyond the signs and symptoms from a structured questionnaire. Additionally, InSilicoVA is designed to work at a population‐level by aggregating probabilities rather than assigning an individual death a single cause, as we have done in this study, to facilitate individual‐level comparisons with physician review. However, given that the probability for the most probable CoD from InSilicoVA is over 0.97 in over half the cases, the individual‐level comparison seems valid.

This study has highlighted challenges in tracking CoD in Malawi, and across the African region, as an increasing percentage of deaths occur in older people where CoD is harder to ascertain from VA. In the short term, we recommend cautious use of VA, particularly when used in conjunction with algorithms, focusing on broad groupings of CoDs (e.g., non‐communicable disease or neoplasm) rather than trying to use more specific CoDs (e.g., digestive neoplasm). In the longer term, and while VA remains an important tool in many settings, we recommend several adaptations to the existing tools to improve CoD assignment accuracy: first, a two‐stage CoD assignment, with algorithms used initially to assign straightforward CoDs (e.g., injuries and infectious diseases with distinct symptoms patterns) and physicians then used to assign CoD for more complex cases where additional data from the narrative is likely to facilitate assignment (e.g., for neoplasms). Second, there is a limited set of questions in the World Health Organization VA tool on medical diagnoses; this should be expanded to allow the reports of any medical diagnoses from a doctor. Finally, it is essential that there is a continuation of the collection of rich HDSS data with VA and other health information as with Karonga HDSS, to provide more reference deaths to enable the improvement of CoD attribution for all VA methods.

## FUNDING INFORMATION

Funding for the study was provided by the National Institute of Child Health and Human Development grant 1R01HD086227.

## Supporting information


**Data S1.** Supporting Information.
