# Supplementary material for: Evaluating the performance of verbal autopsy for assigning cause of death in older adults: A population‐based cohort study in Karonga, Malawi
Source: Trop Med Int Health. 2025 May 13;30(7):652–61. doi: 10.1111/tmi.14120 (PMC12213313; doi:10.1111/tmi.14120)
Supplement: Supplementary file 1 — Data S1. Supporting Information. [file TMI-30-652-s001.docx]

# Supplementary materials

Title

Evaluating the performance of verbal autopsy for assigning cause of death in older adults: a population-based cohort study in Karonga, Malawi

# Authors

Milly Marston^1^, Alex Chung^2^, Albert Dube^3^, Estelle McLean^1,3^, Samuel Clark^5^, Amelia Crampin^1,3^ and Clara Calvert^5^

Supplementary Table 1: Cause of death categories available in Karonga physician review data, and broad cause categories created for this study

| **Broad Cause** | **Specific Cause** |
| --- | --- |
| Communicable Disease | Unspecifiable/other |
| Communicable Disease | Acute febrile illness (Unspec/other) |
| Communicable Disease | Malaria |
| Communicable Disease | Meningitis |
| Communicable Disease | Pneumonia |
| Communicable Disease | Diarrhoea with fever |
| Communicable Disease | Sepsis |
| Communicable Disease | Hepatitis |
| Communicable Disease | TB/AIDS (Unspec/other) |
| Communicable Disease | Pulmonary TB |
| Communicable Disease | AIDS |
| Communicable Disease | Extrapulmonary TB |
| Communicable Disease | Diarrhoeal disease without fever |
| Communicable Disease | Rabies |
| Non-Communicable Disease | Unspecifiable/other |
| Non-Communicable Disease | Cardiovascular disorder (unspec/other) |
| Non-Communicable Disease | Hypertension |
| Non-Communicable Disease | Congestive heart disease |
| Non-Communicable Disease | Ischaemic heart disease |
| Non-Communicable Disease | Cerebro vascular disease |
| Non-Communicable Disease | Respiratory disorder (unspec/other) |
| Non-Communicable Disease | Chronic obstructive pulmonary disease |
| Non-Communicable Disease | Asthma |
| Non-Communicable Disease | Gastro intestinal disorder (unspec/other) |
| Non-Communicable Disease | Peptic ulcer disease |
| Non-Communicable Disease | Liver cirrhosis |
| Non-Communicable Disease | Acute abdomen including obstruction |
| Non-Communicable Disease | Central nervous system disorder (unspec/other) |
| Non-Communicable Disease | Mental/behavioural disorder |
| Non-Communicable Disease | Epilepsy |
| Non-Communicable Disease | Endocrine disorders (unspec/other) |
| Non-Communicable Disease | Diabetes |
| Non-Communicable Disease | Neoplasm (unspec/other) |
| Non-Communicable Disease | Neoplasm - breast |
| Non-Communicable Disease | Neoplasm - cervix/uterus |
| Non-Communicable Disease | Neoplasm - liver |
| Non-Communicable Disease | Neoplasm - GI exc liver |
| Non-Communicable Disease | Neoplasm - lung |
| Non-Communicable Disease | Neoplasm - oral |
| Non-Communicable Disease | Genito urinary disorders (unspec/other) |
| Non-Communicable Disease | Kidney disorder |
| Non-Communicable Disease | Anaemia (unspec/other) |
| Non-Communicable Disease | Anaemia (Caused by chronic CD) |
| Non-Communicable Disease | Nutritional disorder (unspec/other) |
| Non-Communicable Disease | Malnutrition |
| Direct obstetric causes | Unspecifiable/other |
| Direct obstetric causes | Abortion |
| Direct obstetric causes | Eclampsia |
| Direct obstetric causes | Ante/postpartum haemorrhage |
| Direct obstetric causes | Obstructed labour |
| Direct obstetric causes | Puerperal sepsis |
| Direct obstetric causes | Anaemia in pregnancy |
| External | Unspecifiable/other |
| External | Transport |
| External | Fall |
| External | Drowning/submersion |
| External | Exposure to smoke/fire/flames |
| External | Poisoning/exposure to noxious substance |
| External | Use of weapon |
| External | Hanging |

Supplementary Table 2: Cause of death categories available in InSilicoVA output, and broad cause categories created for this study

| **Broad Cause** | **Specific Cause** |
| --- | --- |
| Communicable Disease | Sepsis (non-obstetric) |
| Communicable Disease | Acute resp infect incl pneumonia |
| Communicable Disease | HIV/AIDS related death |
| Communicable Disease | Diarrhoeal diseases |
| Communicable Disease | Malaria |
| Communicable Disease | Meningitis and encephalitis |
| Communicable Disease | Tetanus |
| Communicable Disease | Pulmonary tuberculosis |
| Communicable Disease | Haemorrhagic fever (non-dengue) |
| Communicable Disease | Dengue fever |
| Communicable Disease | Other and unspecified infect dis |
| Non-Communicable Disease | Oral neoplasms |
| Non-Communicable Disease | Digestive neoplasms |
| Non-Communicable Disease | Respiratory neoplasms |
| Non-Communicable Disease | Breast neoplasms |
| Non-Communicable Disease | Reproductive neoplasms MF |
| Non-Communicable Disease | Other and unspecified neoplasms |
| Non-Communicable Disease | Severe anaemia |
| Non-Communicable Disease | Severe malnutrition |
| Non-Communicable Disease | Diabetes mellitus |
| Non-Communicable Disease | Acute cardiac disease |
| Non-Communicable Disease | Stroke |
| Non-Communicable Disease | Other and unspecified cardiac dis |
| Non-Communicable Disease | Chronic obstructive pulmonary dis |
| Non-Communicable Disease | Asthma |
| Non-Communicable Disease | Acute abdomen |
| Non-Communicable Disease | Liver cirrhosis |
| Non-Communicable Disease | Renal failure |
| Non-Communicable Disease | Epilepsy |
| Non-Communicable Disease | Other and unspecified NCD |
| Direct obstetric causes | Ectopic pregnancy |
| Direct obstetric causes | Abortion-related death |
| Direct obstetric causes | Pregnancy-induced hypertension |
| Direct obstetric causes | Obstetric haemorrhage |
| Direct obstetric causes | Obstructed labour |
| Direct obstetric causes | Pregnancy-related sepsis |
| Direct obstetric causes | Anaemia of pregnancy |
| Direct obstetric causes | Ruptured uterus |
| External | Road traffic accident |
| External | Accid fall |
| External | Accid drowning and submersion |
| External | Accid poisoning & noxious subs |
| External | Intentional self-harm |
| External | Assault |
| External | Exposure to force of nature |
| External | Other and unspecified external CoD |
| Indeterminate | Indeterminate |

Supplementary Table 3: Harmonised cause of death categorisation to allow the most detail comparison between the physician review and InsilicoVA assigned cause of death

| **Broad Cause** | **Harmonised Cause** | **Physician Cause** | **InsilicoVA Cause** |
| --- | --- | --- | --- |
| *Communicable Disease* | |  |  |
|  | Sepsis (non-obstetric) | CD-Sepsis | Sepsis (non-obstetric) |
|  | Acute resp infect incl pneumonia | CD-Pneumonia | Acute resp infect incl pneumonia |
|  | Diarrhoeal diseases | CD-Diarrhoeal disease without fever | Diarrhoeal diseases |
|  | Malaria | CD-Malaria | Malaria |
|  | Meningitis and encephalitis | CD-Meningitis | Meningitis and encephalitis |
|  | Other and unspecified infect dis | CD-Unspecifiable/other |  |
|  | Other and unspecified infect dis | CD-Acute febrile illness (Unspec/other) |  |
|  | Other and unspecified infect dis | CD-Diarrhoea with fever |  |
|  | Other and unspecified infect dis | CD-Hepatitis |  |
|  | Other and unspecified infect dis | CD-Rabies |  |
|  | Other and unspecified infect dis |  | Tetanus |
|  | Other and unspecified infect dis |  | Haemorrhagic fever (non-dengue) |
|  | Other and unspecified infect dis |  | Dengue fever |
|  | Other and unspecified infect dis |  | Other and unspecified infect dis |
|  | TB/HIV/AIDS | CD-TB/AIDS (Unspec/other) |  |
|  | TB/HIV/AIDS | CD-Pulmonary TB | Pulmonary tuberculosis |
|  | TB/HIV/AIDS | CD-AIDS |  |
|  | TB/HIV/AIDS | CD-Extrapulmonary TB |  |
|  | TB/HIV/AIDS |  | HIV/AIDS related death |
| *Non-Communicable Disease* | |  |  |
|  | Oral neoplasms | NCD-Neoplasm - oral | Oral neoplasms |
|  | Digestive neoplasms | NCD-Neoplasm - GI exc liver | Digestive neoplasms |
|  | Respiratory neoplasms | NCD-Neoplasm - lung | Respiratory neoplasms |
|  | Breast neoplasms | NCD-Neoplasm - breast | Breast neoplasms |
|  | Reproductive neoplasms MF | NCD-Neoplasm - cervix/uterus | Reproductive neoplasms MF |
|  |  | 372 |  |
|  | Other and unspecified neoplasms | NCD-Neoplasm (unspec/other) |  |
|  | Other and unspecified neoplasms | NCD-Neoplasm - liver |  |
|  | Other and unspecified neoplasms |  | Other and unspecified neoplasms |
|  | Severe anaemia | NCD-Anaemia (unspec/other) | Severe anaemia |
|  | Severe anaemia | NCD-Anaemia (Caused by chronic CD) |  |
|  | Severe malnutrition | NCD-Nutritional disorder (unspec/other) |  |
|  | Severe malnutrition | NCD-Malnutrition | Severe malnutrition |
|  | Diabetes mellitus | NCD-Endocrine disorders (unspec/other) |  |
|  | Diabetes mellitus | NCD-Diabetes | Diabetes mellitus |
|  | Unspecified cardiac dis/Stroke | NCD-Cardiovascular disorder (unspec/other) |  |
|  | Unspecified cardiac dis/Stroke | NCD-Hypertension |  |
|  | Unspecified cardiac dis/Stroke | NCD-Congestive heart disease |  |
|  | Unspecified cardiac dis/Stroke | NCD-Ischaemic heart disease |  |
|  | Unspecified cardiac dis/Stroke | NCD-Cerebro vascular disease |  |
|  | Unspecified cardiac dis/Stroke |  | Acute cardiac disease |
|  | Unspecified cardiac dis/Stroke |  | Stroke |
|  | Unspecified cardiac dis/Stroke |  | Other and unspecified cardiac dis |
|  | Asthma | NCD-Asthma | Asthma |
|  | Acute abdomen | NCD-Acute abdomen including obstruction | Acute abdomen |
|  | Liver cirrhosis | NCD-Liver cirrhosis | Liver cirrhosis |
|  | Renal failure | NCD-Kidney disorder | Renal failure |
|  | Epilepsy | NCD-Epilepsy | Epilepsy |
|  | Other and unspecified NCD | NCD-Unspecifiable/other |  |
|  | Other and unspecified NCD | NCD-Respiratory disorder (unspec/other) |  |
|  | Other and unspecified NCD | NCD-Chronic obstructive pulmonary disease |  |
|  | Other and unspecified NCD | NCD-Gastro intestinal disorder (unspec/other) |  |
|  | Other and unspecified NCD | NCD-Peptic ulcer disease |  |
|  | Other and unspecified NCD | NCD-Central nervous system disorder (unspec/other) |  |
|  | Other and unspecified NCD | NCD-Mental/behavioural disorder |  |
|  | Other and unspecified NCD | NCD-Genito urinary disorders (unspec/other) |  |
|  | Other and unspecified NCD |  | Chronic obstructive pulmonary dis |
|  | Other and unspecified NCD |  | Other and unspecified NCD |
| *External* | |  |  |
|  | Accid fall | EXT-Fall | Accid fall |
|  | Accid drowning and submersion | EXT-Drowning/submersion | Accid drowning and submersion |
|  | Accid expos to smoke fire & flame | EXT-Exposure to smoke/fire/flames | Exposure to smoke/fire/flames |
|  | Accid poisoning & noxious subs | EXT-Poisoning/exposure to noxious substance | Accid poisoning & noxious subs |
|  | Other and unspecified external CoD | EXT-Unspecifiable/other |  |
|  | Other and unspecified external CoD | EXT-Use of weapon |  |
|  | Other and unspecified external CoD | EXT-Hanging |  |
|  | Other and unspecified external CoD |  | Intentional self-harm |
|  | Other and unspecified external CoD |  | Assault |
|  | Other and unspecified external CoD |  | Exposure to force of nature |
|  | Other and unspecified external CoD |  | Other and unspecified external CoD |
|  | Transport Accident | EXT-Transport | Road traffic accident |
| *Direct Obstetric Causes* | |  |  |
|  | Abortion-related death | MATERNAL-Abortion | Abortion-related death |
|  | Pregnancy-induced hypertension | MATERNAL-Eclampsia | Pregnancy-induced hypertension |
|  | Obstetric haemorrhage | MATERNAL-Ante/postpartum haemorrhage | Obstetric haemorrhage |
|  | Obstructed labour | MATERNAL-Obstructed labour | Obstructed labour |
|  | Pregnancy-related sepsis | MATERNAL-Puerperal sepsis | Pregnancy-related sepsis |
|  | Anaemia of pregnancy | MATERNAL-Anaemia in pregnancy | Anaemia of pregnancy |
|  | Other and unspecified maternal CoD | MATERNAL-Unspecifiable/other |  |
|  | Other and unspecified maternal CoD |  | Ectopic pregnancy |
|  | Other and unspecified maternal CoD |  | Ruptured uterus |
| *Indeterminate* | |  |  |
|  | Indeterminate |  | Indeterminate |

CD=Communicable Disease; NCD=Non Communicable Disease; Ext=External

Supplementary Figure 1: percentage of cases that have underlying, direct or contributory cause of death assigned by physcians from 2012, by sex and broad age group

Supplementary Table 4: Probability assigned, by InsilicoVA, to the most probable cause of death by age, sex and percentile

|  | Men | | |  | Women | | |
| --- | --- | --- | --- | --- | --- | --- | --- |
|  | 25% | Median | 60% |  | 25% | Median | 60% |
| 15-29 | 0.65 | 0.99 | 0.99 |  | 0.82 | 0.95 | 0.99 |
| 30-49 | 0.80 | 0.99 | 0.99 |  | 0.84 | 0.99 | 1.00 |
| 50-59 | 0.78 | 0.98 | 1.00 |  | 0.80 | 0.98 | 0.99 |
| 60-79 | 0.77 | 0.97 | 0.99 |  | 0.78 | 0.96 | 0.98 |
| 80+ | 0.74 | 0.93 | 0.98 |  | 0.70 | 0.93 | 0.98 |
| Total | 0.77 | 0.98 | 0.99 |  | 0.79 | 0.97 | 0.99 |

Supplementary Table 5: Number and percentage of deaths where the most probable cause of death assigned by InsilicoVA had a probability over 90% (excluding external cause of death), by age group and sex

|  | Men  N=1071* | |  | Women  N=1166* | |
| --- | --- | --- | --- | --- | --- |
| 15-29 | 70 | 68.0% |  | 102 | 64.2% |
| 30-49 | 258 | 67.4% |  | 221 | 66.8% |
| 50-59 | 86 | 63.7% |  | 68 | 63.0% |
| 60-79 | 179 | 63.3% |  | 212 | 59.6% |
| 80+ | 106 | 60.2% |  | 124 | 52.5% |
| Total | 699 | 64.7% |  | 727 | 61.1% |

*Excluding external causes of death (men n=137, women n=27)

Supplementary Table 6: Percentages of broad cause of death and specific harmonised cause of death disagreement between InSilicoVA and physician review by sex for 2012-2017

| **Age Group** | **Broad Cause** | |  |
| --- | --- | --- | --- |
|  | **Male** | **Female** | **Total** |
|  | **(n=471)** | **(n=426)** | **(n=897)** |
| **15-29** | 16.90 | 32.70 | 23.90 |
| **30-49** | 28.00 | 17.20 | 24.10 |
| **50-59** | 36.40 | 18.40 | 29.00 |
| **60-79** | 32.80 | 21.70 | 26.70 |
| **80+** | 22.00 | 35.90 | 29.80 |
| **Total** | 27.60 | 25.35 | 26.50 |
| * Men Chi2 p-value=0.068 | | |  |
| * Women Chi2 p-value=0.013 | | |  |

Supplementary Table 7: Percentages of broad cause of death and specific harmonised cause of death disagreement between InSilicoVA and physician review by sex for 2012-2017, allowing a match to be any form underlying, direct or contributory cause of death to match.

| **Age Group** | **Broad Cause** | |  |
| --- | --- | --- | --- |
|  | **Male** | **Female** | **Total** |
|  | **(n=471)** | **(n=426)** | **(n=897)** |
| **15-29** | 13.90 | 30.80 | 21.40 |
| **30-49** | 22.00 | 13.80 | 18.99 |
| **50-59** | 29.10 | 13.16 | 22.58 |
| **60-79** | 25.20 | 17.48 | 20.99 |
| **80+** | 15.90 | 31.13 | 24.47 |
| **Total** | 21.44 | 21.36 | 21.40 |
| * Men Chi2 p-value=0.153 | | |  |
| * Women Chi2 p-value=0.006 | | |  |

Supplementary Table 8: Percentages of non-matching broad cause of death between InSilicoVA and physician review by sex (N=77 for men and N=76 for women), for 2012-2017

| **InSilicoVA** | | **Physician review** | | | |  |
| --- | --- | --- | --- | --- | --- | --- |
|  |  | Communicable Disease | Non-Communicable Disease | External | Indeterminate | **Total** |
|  |  | n (%) | n (%) | n (%) | n (%) | n (%) |
| **Men** | |  |  |  |  |  |
|  | Communicable Disease | - | 24 (33.2%) | 5 (6.5%) | 4(5.2%) | 33 (42.9%) |
|  | Non-Communicable Disease | 18 (23.4%) | - | 4 (5.2%) | 13(16.9%) | 35 (45.5%) |
|  | External | 0 (0.0%) | 6 (7.8%) | - | 2(2.6%) | 8(10.4%) |
|  | Indeterminate | 0 (0.0%) | 1 (1.3%) | 0 (0.0%) | - | 1 (1.3%) |
|  | **Total** | 18 (23.4%) | 31 (40.3%) | 9 (11.7%) | 19 (24.7%) | 77 (100%) |
| **Women** | |  |  |  |  |  |
|  | Communicable Disease | - | 23 (30.8%) | 1(0.9%) | 9 (10.1%) | 33 (43.4%) |
|  | Non-Communicable Disease | 14 (25.6%) | - | 0 (0.0%) | 21 (23.8%) | 35 (46.1%) |
|  | External | 0 (0.4%) | 4 (2.2%) | - | 0 (0.4%) | 4 (5.3%) |
|  | Indeterminate | 2 (2.2%) | 2 (3.5%) | 0 (0.0%) | - | 4 (5.3%) |
|  | **Total** | 16 (28.2%) | 29 (36.6%) | 1 (0.9%) | 30 (34.4%) | 76 (100%) |

Supplementary Table 9: Percentages of non-matching broad cause of death between InSilicoVA and physician review by sex (N=59 for men and N=63 for women), for 2012-2017, allowing a match to be any form underlying, direct or contributory cause of death

| **InSilicoVA** | | **Physician review** | | | |  |
| --- | --- | --- | --- | --- | --- | --- |
|  |  | Communicable Disease | Non-Communicable Disease | External | Indeterminate | **Total** |
|  |  | n (%) | n (%) | n (%) | n (%) | n (%) |
| **Men** | |  |  |  |  |  |
|  | Communicable Disease | - | 19(32.2%) | 3 (5.1%) | 4(6.8%) | 26 (44.1%) |
|  | Non-Communicable Disease | 12 (20.3%) | - | 4 (6.8%) | 11(18.6%) | 27 (45.8%) |
|  | External | 0 (0.0%) | 4 (6.8%) | - | 2(2.7%) | 5(8.5%) |
|  | Indeterminate | 0 (0.0%) | 1 (1.69%) | 0 (0.0%) | - | 1 (1.7%) |
|  | **Total** | 12 (20.3%) | 24 (40.7%) | 7 (11.9%) | 16 (27.2%) | 59 (100%) |
| **Women** | |  |  |  |  |  |
|  | Communicable Disease | - | 19 (30.2%) | 1(1.6%) | 9 (14.3%) | 29 (46.0%) |
|  | Non-Communicable Disease | 9 (14.3%) | - | 0 (0.0%) | 18 (28.6%) | 27 (42.9%) |
|  | External | 0 (0.4%) | 3 (4.8%) | - | 0 (0.0%) | 3(4.8%) |
|  | Indeterminate | 2 (3.2%) | 2 (3.2%) | 0 (0.0%) | - | 4 (6.4%) |
|  | **Total** | 11 (17.5%) | 24 (38.1%) | 1 (1.6%) | 27 (42.9%) | 63 (100%) |

Supplementary Table 10: Comparison of harmonised cause of death assignment between physician review underlying cause of death and InSilicoVA for deaths to adults aged 50 and over

|  |  | Physician Cause of Death | | | | | | | | | | | | | | | | | | | | | | | | | | | | | |  |
| --- | --- | --- | --- | --- | --- | --- | --- | --- | --- | --- | --- | --- | --- | --- | --- | --- | --- | --- | --- | --- | --- | --- | --- | --- | --- | --- | --- | --- | --- | --- | --- | --- |
|  |  | Sepsis (non-obstetric | Acute resp infect inc | Diarrhoeal diseases | Malaria | Meningitis and enceph | Oral neoplasms | Digestive neoplasms | Respiratory neoplasms | Breast neoplasms | Reproductive neoplasm | Other and unspecified | Severe anaemia | Severe malnutrition | Diabetes mellitus | Unspecified cardiac d | Asthma | Acute abdomen | Liver cirrhosis | Renal failure | Epilepsy | Accid fall | Accid drowning | Accid poisoning & nox | Other and unspecified | Other and unspecified | TB/HIV/AIDS | Other and unspecified NCD | Transport Accident | Indeterminate | Total | |
| InSilicoVA Cause of death | Sepsis (non-obstetric | 0 | 0 | 0 | 0 | 0 | 0 | 0 | 0 | 0 | 0 | 0 | 0 | 0 | 0 | 1 | 0 | 0 | 0 | 0 | 1 | 0 | 0 | 0 | 0 | 0 | 0 | 0 | 0 | 0 | **2** | |
|  | Acute resp infect inc | 2 | 23 | 1 | 4 | 1 | 0 | 0 | 0 | 0 | 0 | 2 | 1 | 0 | 6 | 36 | 10 | 0 | 0 | 0 | 0 | 0 | 0 | 0 | 1 | 4 | 9 | 8 | 0 | 6 | **114** | |
|  | Diarrhoeal diseases | 0 | 0 | 0 | 1 | 0 | 0 | 0 | 0 | 0 | 0 | 0 | 0 | 0 | 1 | 0 | 0 | 0 | 0 | 0 | 0 | 0 | 0 | 0 | 0 | 1 | 2 | 1 | 0 | 0 | **6** | |
|  | Malaria | 0 | 4 | 0 | 3 | 0 | 0 | 0 | 0 | 0 | 0 | 0 | 0 | 0 | 0 | 3 | 0 | 0 | 0 | 0 | 0 | 0 | 0 | 0 | 0 | 2 | 1 | 0 | 0 | 1 | **14** | |
|  | Meningitis and enceph | 0 | 0 | 0 | 0 | 2 | 0 | 0 | 0 | 0 | 0 | 0 | 0 | 0 | 0 | 0 | 0 | 0 | 0 | 0 | 0 | 0 | 0 | 0 | 0 | 1 | 0 | 0 | 0 | 0 | **3** | |
|  | Oral neoplasms | 0 | 0 | 0 | 0 | 0 | 0 | 0 | 0 | 0 | 0 | 1 | 0 | 1 | 0 | 1 | 0 | 0 | 0 | 0 | 0 | 0 | 0 | 0 | 0 | 1 | 0 | 0 | 0 | 3 | **7** | |
|  | Digestive neoplasms | 0 | 2 | 6 | 0 | 0 | 0 | 9 | 1 | 0 | 4 | 18 | 1 | 0 | 0 | 16 | 0 | 5 | 16 | 1 | 0 | 0 | 0 | 0 | 0 | 8 | 17 | 33 | 0 | 5 | **142** | |
|  | Respiratory neoplasms | 0 | 1 | 0 | 1 | 0 | 0 | 2 | 2 | 0 | 0 | 3 | 0 | 1 | 0 | 16 | 3 | 0 | 0 | 0 | 0 | 0 | 0 | 0 | 0 | 1 | 5 | 5 | 0 | 5 | **45** | |
|  | Breast neoplasms | 0 | 0 | 0 | 0 | 0 | 0 | 0 | 0 | 2 | 0 | 0 | 0 | 0 | 0 | 1 | 0 | 0 | 0 | 0 | 0 | 0 | 0 | 0 | 0 | 0 | 0 | 0 | 0 | 0 | **3** | |
|  | Reproductive neoplasm | 0 | 0 | 0 | 0 | 0 | 0 | 3 | 0 | 0 | 32 | 3 | 0 | 0 | 0 | 0 | 0 | 0 | 0 | 0 | 0 | 0 | 0 | 0 | 0 | 0 | 1 | 0 | 0 | 0 | **39** | |
|  | Other and unspecified | 0 | 0 | 0 | 0 | 0 | 0 | 7 | 0 | 2 | 1 | 4 | 0 | 0 | 0 | 4 | 0 | 0 | 1 | 0 | 0 | 0 | 0 | 0 | 0 | 0 | 1 | 6 | 0 | 4 | **30** | |
|  | Severe anaemia | 0 | 0 | 0 | 1 | 0 | 0 | 0 | 0 | 0 | 0 | 0 | 2 | 1 | 0 | 2 | 0 | 0 | 0 | 0 | 0 | 0 | 0 | 0 | 0 | 0 | 0 | 0 | 0 | 1 | **7** | |
|  | Severe malnutrition | 0 | 0 | 0 | 0 | 0 | 0 | 0 | 0 | 0 | 0 | 0 | 0 | 0 | 0 | 0 | 0 | 0 | 0 | 0 | 0 | 0 | 0 | 0 | 0 | 1 | 2 | 0 | 0 | 0 | **3** | |
|  | Diabetes mellitus | 0 | 0 | 0 | 2 | 0 | 0 | 0 | 0 | 0 | 0 | 0 | 0 | 0 | 15 | 6 | 0 | 0 | 0 | 0 | 0 | 0 | 0 | 0 | 0 | 0 | 0 | 1 | 0 | 1 | **25** | |
|  | Unspecified cardiac disease | 0 | 9 | 0 | 2 | 0 | 0 | 3 | 0 | 2 | 2 | 5 | 5 | 0 | 14 | 216 | 0 | 0 | 0 | 0 | 0 | 0 | 1 | 3 | 0 | 4 | 10 | 20 | 0 | 17 | **313** | |
|  | Asthma | 0 | 0 | 0 | 0 | 0 | 0 | 0 | 0 | 0 | 0 | 0 | 0 | 0 | 0 | 1 | 5 | 0 | 0 | 0 | 0 | 0 | 0 | 0 | 0 | 0 | 0 | 0 | 0 | 1 | **7** | |
|  | Acute abdomen | 1 | 1 | 1 | 2 | 0 | 0 | 1 | 0 | 0 | 0 | 1 | 0 | 0 | 6 | 11 | 0 | 15 | 2 | 0 | 0 | 0 | 0 | 2 | 0 | 8 | 5 | 18 | 0 | 12 | **86** | |
|  | Liver cirrhosis | 0 | 0 | 0 | 0 | 0 | 0 | 0 | 0 | 0 | 0 | 0 | 0 | 0 | 0 | 14 | 0 | 1 | 3 | 0 | 1 | 0 | 0 | 0 | 0 | 0 | 3 | 5 | 0 | 2 | **29** | |
|  | Renal failure | 0 | 0 | 0 | 1 | 0 | 0 | 0 | 0 | 0 | 0 | 0 | 0 | 0 | 0 | 4 | 0 | 0 | 0 | 6 | 0 | 0 | 0 | 0 | 0 | 0 | 3 | 2 | 0 | 2 | **18** | |
|  | Epilepsy | 0 | 0 | 0 | 0 | 0 | 0 | 0 | 0 | 0 | 0 | 0 | 0 | 0 | 1 | 0 | 0 | 0 | 0 | 0 | 0 | 0 | 0 | 1 | 0 | 0 | 0 | 0 | 0 | 0 | **2** | |
|  | Accid fall | 0 | 0 | 0 | 0 | 0 | 0 | 0 | 0 | 0 | 0 | 0 | 0 | 0 | 0 | 4 | 0 | 0 | 0 | 0 | 0 | 3 | 0 | 0 | 0 | 0 | 0 | 0 | 0 | 1 | **8** | |
|  | Accid drowning | 0 | 0 | 0 | 0 | 0 | 0 | 0 | 0 | 0 | 0 | 0 | 0 | 0 | 0 | 0 | 0 | 0 | 0 | 0 | 0 | 0 | 0 | 0 | 0 | 0 | 0 | 0 | 0 | 0 | **0** | |
|  | Accid poisoning & nox | 0 | 0 | 0 | 0 | 0 | 0 | 0 | 0 | 0 | 0 | 0 | 0 | 0 | 0 | 0 | 0 | 0 | 0 | 0 | 0 | 0 | 0 | 1 | 0 | 0 | 0 | 0 | 0 | 0 | **1** | |
|  | Other and unspecified external | 1 | 0 | 0 | 0 | 0 | 0 | 0 | 0 | 0 | 0 | 0 | 0 | 0 | 0 | 2 | 0 | 0 | 0 | 0 | 0 | 3 | 2 | 1 | 13 | 0 | 0 | 4 | 0 | 2 | **28** | |
|  | Other and unspecified infect dis | 4 | 10 | 3 | 3 | 2 | 0 | 1 | 0 | 0 | 2 | 2 | 3 | 2 | 5 | 19 | 1 | 4 | 0 | 5 | 0 | 1 | 0 | 1 | 0 | 28 | 13 | 12 | 0 | 15 | **136** | |
|  | TB/HIV/AIDS | 0 | 1 | 1 | 0 | 0 | 0 | 1 | 0 | 0 | 0 | 2 | 0 | 1 | 0 | 5 | 1 | 0 | 0 | 0 | 0 | 0 | 0 | 4 | 0 | 3 | 92 | 3 | 0 | 0 | **114** | |
|  | Other and unspecified NCD | 1 | 1 | 2 | 0 | 0 | 0 | 0 | 0 | 0 | 0 | 0 | 0 | 3 | 2 | 10 | 1 | 0 | 0 | 0 | 0 | 0 | 0 | 0 | 0 | 2 | 2 | 8 | 0 | 7 | **39** | |
|  | Transport Accident | 1 | 0 | 0 | 0 | 0 | 0 | 0 | 0 | 0 | 0 | 0 | 0 | 0 | 3 | 2 | 0 | 0 | 0 | 0 | 0 | 2 | 0 | 0 | 2 | 0 | 0 | 1 | 12 | 1 | **24** | |
|  | Indeterminate | 0 | 0 | 0 | 2 | 0 | 0 | 0 | 0 | 0 | 0 | 1 | 2 | 0 | 2 | 5 | 0 | 0 | 0 | 0 | 0 | 0 | 0 | 2 | 0 | 0 | 3 | 4 | 0 | 10 | **31** | |
|  | **Total** | **10** | **52** | **14** | **22** | **5** | **0** | **27** | **3** | **6** | **41** | **42** | **14** | **9** | **55** | **379** | **21** | **25** | **22** | **12** | **2** | **9** | **3** | **15** | **16** | **64** | **169** | **131** | **12** | **96** | **1276** | |

Supplementary Table 11: Percentage distribution of most probable cause of death for an individual assigned by InsilicoVA by Physician review cause of death for men and women 50 year old and over.

|  |  |  | | Physician Cause of Death | | | | | | | | | | | | | | | | | | | | | | | | | | | | |
| --- | --- | --- | --- | --- | --- | --- | --- | --- | --- | --- | --- | --- | --- | --- | --- | --- | --- | --- | --- | --- | --- | --- | --- | --- | --- | --- | --- | --- | --- | --- | --- | --- |
|  |  | Sepsis (non-obstetric) | Acute resp infect inc | | Diarrhoeal diseases | Malaria | Meningitis and enceph | Oral neoplasms | Digestive neoplasms | Respiratory neoplasms | Breast neoplasms | Reproductive neoplasm | Other and unspecified | Severe anaemia | Severe malnutrition | Diabetes mellitus | Unspecified cardiac disease | Asthma | Acute abdomen | Liver cirrhosis | Renal failure | Epilepsy | Accid fall | Accid drowning | Accid poisoning & nox | Other and unspecified | Other and unspecified | TB/HIV/AIDS | Other and unspecified NCDs | Transport Accident | Indeterminate | **Total** |
| InSilicoVA Cause of Death | Sepsis (non-obstetric) | 0.0 | 0.0 | | 0.0 | 0.0 | 0.0 | - | 0.0 | 0.0 | 0.0 | 0.0 | 0.0 | 0.0 | 0.0 | 0.0 | 0.3 | 0.0 | 0.0 | 0.0 | 0.0 | 50.0 | 0.0 | 0.0 | 0.0 | 0.0 | 0.0 | 0.0 | 0.0 | 0.0 | 0.0 | **0.2** |
|  | Acute resp infect inc | 20.0 | 44.2 | | 7.1 | 18.2 | 20.0 | - | 0.0 | 0.0 | 0.0 | 0.0 | 4.8 | 7.1 | 0.0 | 10.9 | 9.5 | 47.6 | 0.0 | 0.0 | 0.0 | 0.0 | 0.0 | 0.0 | 0.0 | 6.3 | 6.3 | 5.3 | 6.1 | 0.0 | 6.3 | **8.9** |
|  | Diarrhoeal diseases | 0.0 | 0.0 | | 0.0 | 4.5 | 0.0 | - | 0.0 | 0.0 | 0.0 | 0.0 | 0.0 | 0.0 | 0.0 | 1.8 | 0.0 | 0.0 | 0.0 | 0.0 | 0.0 | 0.0 | 0.0 | 0.0 | 0.0 | 0.0 | 1.6 | 1.2 | 0.8 | 0.0 | 0.0 | **0.5** |
|  | Malaria | 0.0 | 7.7 | | 0.0 | 13.6 | 0.0 | - | 0.0 | 0.0 | 0.0 | 0.0 | 0.0 | 0.0 | 0.0 | 0.0 | 0.8 | 0.0 | 0.0 | 0.0 | 0.0 | 0.0 | 0.0 | 0.0 | 0.0 | 0.0 | 3.1 | 0.6 | 0.0 | 0.0 | 1.0 | **1.1** |
|  | Meningitis and enceph | 0.0 | 0.0 | | 0.0 | 0.0 | 40.0 | - | 0.0 | 0.0 | 0.0 | 0.0 | 0.0 | 0.0 | 0.0 | 0.0 | 0.0 | 0.0 | 0.0 | 0.0 | 0.0 | 0.0 | 0.0 | 0.0 | 0.0 | 0.0 | 1.6 | 0.0 | 0.0 | 0.0 | 0.0 | **0.2** |
|  | Oral neoplasms | 0.0 | 0.0 | | 0.0 | 0.0 | 0.0 | - | 0.0 | 0.0 | 0.0 | 0.0 | 2.4 | 0.0 | 11.1 | 0.0 | 0.3 | 0.0 | 0.0 | 0.0 | 0.0 | 0.0 | 0.0 | 0.0 | 0.0 | 0.0 | 1.6 | 0.0 | 0.0 | 0.0 | 3.1 | **0.5** |
|  | Digestive neoplasms | 0.0 | 3.8 | | 42.9 | 0.0 | 0.0 | - | 33.3 | 33.3 | 0.0 | 9.8 | 42.9 | 7.1 | 0.0 | 0.0 | 4.2 | 0.0 | 20.0 | 72.7 | 8.3 | 0.0 | 0.0 | 0.0 | 0.0 | 0.0 | 12.5 | 10.1 | 25.2 | 0.0 | 5.2 | **11.1** |
|  | Respiratory neoplasms | 0.0 | 1.9 | | 0.0 | 4.5 | 0.0 | - | 7.4 | 66.7 | 0.0 | 0.0 | 7.1 | 0.0 | 11.1 | 0.0 | 4.2 | 14.3 | 0.0 | 0.0 | 0.0 | 0.0 | 0.0 | 0.0 | 0.0 | 0.0 | 1.6 | 3.0 | 3.8 | 0.0 | 5.2 | **3.5** |
|  | Breast neoplasms | 0.0 | 0.0 | | 0.0 | 0.0 | 0.0 | - | 0.0 | 0.0 | 33.3 | 0.0 | 0.0 | 0.0 | 0.0 | 0.0 | 0.3 | 0.0 | 0.0 | 0.0 | 0.0 | 0.0 | 0.0 | 0.0 | 0.0 | 0.0 | 0.0 | 0.0 | 0.0 | 0.0 | 0.0 | **0.2** |
|  | Reproductive neoplasm | 0.0 | 0.0 | | 0.0 | 0.0 | 0.0 | - | 11.1 | 0.0 | 0.0 | 78.0 | 7.1 | 0.0 | 0.0 | 0.0 | 0.0 | 0.0 | 0.0 | 0.0 | 0.0 | 0.0 | 0.0 | 0.0 | 0.0 | 0.0 | 0.0 | 0.6 | 0.0 | 0.0 | 0.0 | **3.1** |
|  | Other and unspecified | 0.0 | 0.0 | | 0.0 | 0.0 | 0.0 | - | 25.9 | 0.0 | 33.3 | 2.4 | 9.5 | 0.0 | 0.0 | 0.0 | 1.1 | 0.0 | 0.0 | 4.5 | 0.0 | 0.0 | 0.0 | 0.0 | 0.0 | 0.0 | 0.0 | 0.6 | 4.6 | 0.0 | 4.2 | **2.4** |
|  | Severe anaemia | 0.0 | 0.0 | | 0.0 | 4.5 | 0.0 | - | 0.0 | 0.0 | 0.0 | 0.0 | 0.0 | 14.3 | 11.1 | 0.0 | 0.5 | 0.0 | 0.0 | 0.0 | 0.0 | 0.0 | 0.0 | 0.0 | 0.0 | 0.0 | 0.0 | 0.0 | 0.0 | 0.0 | 1.0 | **0.5** |
|  | Severe malnutrition | 0.0 | 0.0 | | 0.0 | 0.0 | 0.0 | - | 0.0 | 0.0 | 0.0 | 0.0 | 0.0 | 0.0 | 0.0 | 0.0 | 0.0 | 0.0 | 0.0 | 0.0 | 0.0 | 0.0 | 0.0 | 0.0 | 0.0 | 0.0 | 1.6 | 1.2 | 0.0 | 0.0 | 0.0 | **0.2** |
|  | Diabetes mellitus | 0.0 | 0.0 | | 0.0 | 9.1 | 0.0 | - | 0.0 | 0.0 | 0.0 | 0.0 | 0.0 | 0.0 | 0.0 | 27.3 | 1.6 | 0.0 | 0.0 | 0.0 | 0.0 | 0.0 | 0.0 | 0.0 | 0.0 | 0.0 | 0.0 | 0.0 | 0.8 | 0.0 | 1.0 | **2.0** |
|  | Unspecified cardiac disease | 0.0 | 17.3 | | 0.0 | 9.1 | 0.0 | - | 11.1 | 0.0 | 33.3 | 4.9 | 11.9 | 35.7 | 0.0 | 25.5 | 57.0 | 0.0 | 0.0 | 0.0 | 0.0 | 0.0 | 0.0 | 33.3 | 20.0 | 0.0 | 6.3 | 5.9 | 15.3 | 0.0 | 17.7 | **24.5** |
|  | Asthma | 0.0 | 0.0 | | 0.0 | 0.0 | 0.0 | - | 0.0 | 0.0 | 0.0 | 0.0 | 0.0 | 0.0 | 0.0 | 0.0 | 0.3 | 23.8 | 0.0 | 0.0 | 0.0 | 0.0 | 0.0 | 0.0 | 0.0 | 0.0 | 0.0 | 0.0 | 0.0 | 0.0 | 1.0 | **0.5** |
|  | Acute abdomen | 10.0 | 1.9 | | 7.1 | 9.1 | 0.0 | - | 3.7 | 0.0 | 0.0 | 0.0 | 2.4 | 0.0 | 0.0 | 10.9 | 2.9 | 0.0 | 60.0 | 9.1 | 0.0 | 0.0 | 0.0 | 0.0 | 13.3 | 0.0 | 12.5 | 3.0 | 13.7 | 0.0 | 12.5 | **6.7** |
|  | Liver cirrhosis | 0.0 | 0.0 | | 0.0 | 0.0 | 0.0 | - | 0.0 | 0.0 | 0.0 | 0.0 | 0.0 | 0.0 | 0.0 | 0.0 | 3.7 | 0.0 | 4.0 | 13.6 | 0.0 | 50.0 | 0.0 | 0.0 | 0.0 | 0.0 | 0.0 | 1.8 | 3.8 | 0.0 | 2.1 | **2.3** |
|  | Renal failure | 0.0 | 0.0 | | 0.0 | 4.5 | 0.0 | - | 0.0 | 0.0 | 0.0 | 0.0 | 0.0 | 0.0 | 0.0 | 0.0 | 1.1 | 0.0 | 0.0 | 0.0 | 50.0 | 0.0 | 0.0 | 0.0 | 0.0 | 0.0 | 0.0 | 1.8 | 1.5 | 0.0 | 2.1 | **1.4** |
|  | Epilepsy | 0.0 | 0.0 | | 0.0 | 0.0 | 0.0 | - | 0.0 | 0.0 | 0.0 | 0.0 | 0.0 | 0.0 | 0.0 | 1.8 | 0.0 | 0.0 | 0.0 | 0.0 | 0.0 | 0.0 | 0.0 | 0.0 | 6.7 | 0.0 | 0.0 | 0.0 | 0.0 | 0.0 | 0.0 | **0.2** |
|  | Accid fall | 0.0 | 0.0 | | 0.0 | 0.0 | 0.0 | - | 0.0 | 0.0 | 0.0 | 0.0 | 0.0 | 0.0 | 0.0 | 0.0 | 1.1 | 0.0 | 0.0 | 0.0 | 0.0 | 0.0 | 33.3 | 0.0 | 0.0 | 0.0 | 0.0 | 0.0 | 0.0 | 0.0 | 1.0 | **0.6** |
|  | Accid drowning | 0.0 | 0.0 | | 0.0 | 0.0 | 0.0 | - | 0.0 | 0.0 | 0.0 | 0.0 | 0.0 | 0.0 | 0.0 | 0.0 | 0.0 | 0.0 | 0.0 | 0.0 | 0.0 | 0.0 | 0.0 | 0.0 | 0.0 | 0.0 | 0.0 | 0.0 | 0.0 | 0.0 | 0.0 | **0.0** |
|  | Accid poisoning & nox | 0.0 | 0.0 | | 0.0 | 0.0 | 0.0 | - | 0.0 | 0.0 | 0.0 | 0.0 | 0.0 | 0.0 | 0.0 | 0.0 | 0.0 | 0.0 | 0.0 | 0.0 | 0.0 | 0.0 | 0.0 | 0.0 | 6.7 | 0.0 | 0.0 | 0.0 | 0.0 | 0.0 | 0.0 | **0.1** |
|  | Other and unspecified external | 10.0 | 0.0 | | 0.0 | 0.0 | 0.0 | - | 0.0 | 0.0 | 0.0 | 0.0 | 0.0 | 0.0 | 0.0 | 0.0 | 0.5 | 0.0 | 0.0 | 0.0 | 0.0 | 0.0 | 33.3 | 66.7 | 6.7 | 81.3 | 0.0 | 0.0 | 3.1 | 0.0 | 2.1 | **2.2** |
|  | Other and unspecified infect disease | 40.0 | 19.2 | | 21.4 | 13.6 | 40.0 | - | 3.7 | 0.0 | 0.0 | 4.9 | 4.8 | 21.4 | 22.2 | 9.1 | 5.0 | 4.8 | 16.0 | 0.0 | 41.7 | 0.0 | 11.1 | 0.0 | 6.7 | 0.0 | 43.8 | 7.7 | 9.2 | 0.0 | 15.6 | **10.7** |
|  | TB/HIV/AIDS | 0.0 | 1.9 | | 7.1 | 0.0 | 0.0 | - | 3.7 | 0.0 | 0.0 | 0.0 | 4.8 | 0.0 | 11.1 | 0.0 | 1.3 | 4.8 | 0.0 | 0.0 | 0.0 | 0.0 | 0.0 | 0.0 | 26.7 | 0.0 | 4.7 | 54.4 | 2.3 | 0.0 | 0.0 | **8.9** |
|  | Other and unspecified NCD | 10.0 | 1.9 | | 14.3 | 0.0 | 0.0 | - | 0.0 | 0.0 | 0.0 | 0.0 | 0.0 | 0.0 | 33.3 | 3.6 | 2.6 | 4.8 | 0.0 | 0.0 | 0.0 | 0.0 | 0.0 | 0.0 | 0.0 | 0.0 | 3.1 | 1.2 | 6.1 | 0.0 | 7.3 | **3.1** |
|  | Transport Accident | 10.0 | 0.0 | | 0.0 | 0.0 | 0.0 | - | 0.0 | 0.0 | 0.0 | 0.0 | 0.0 | 0.0 | 0.0 | 5.5 | 0.5 | 0.0 | 0.0 | 0.0 | 0.0 | 0.0 | 22.2 | 0.0 | 0.0 | 12.5 | 0.0 | 0.0 | 0.8 | 100 | 1.0 | **1.9** |
|  | Indeterminate | 0.0 | 0.0 | | 0.0 | 9.1 | 0.0 | - | 0.0 | 0.0 | 0.0 | 0.0 | 2.4 | 14.3 | 0.0 | 3.6 | 1.3 | 0.0 | 0.0 | 0.0 | 0.0 | 0.0 | 0.0 | 0.0 | 13.3 | 0.0 | 0.0 | 1.8 | 3.1 | 0.0 | 10.4 | **2.4** |
|  | **Total** | **100** | **100** | | **100** | **100** | **100** | **-** | **100** | **100** | **100** | **100** | **100** | **100** | **100** | **100** | **100** | **100** | **100** | **100** | **100** | **100** | **100** | **100** | **100** | **100** | **100** | **100** | **100** | **100** | **100** | **100** |

Supplementary Table 12: Distribution of Karonga specific physician assigned underlying cause of death for those who were assigned digestive neoplasm by InSilicoVA

| **Underlying Cause of Death** | **Number assigned to digestive neoplasm in InsilicoVA** | |
| --- | --- | --- |
|  | **n** | **%** |
| No significant pathology that would exp | 2 | 1.4 |
| Multiple significant pathology, single | 3 | 2.1 |
| CD-Unspecifiable/other | 3 | 2.1 |
| CD-Acute febrile illness (Unspec/other) | 2 | 1.4 |
| CD-Pneumonia | 2 | 1.4 |
| CD-Diarrhoea with fever | 2 | 1.4 |
| CD-Hepatitis | 1 | 0.7 |
| CD-AIDS | 14 | 9.9 |
| CD-Extrapulmonary TB | 3 | 2.1 |
| CD-Diarrhoeal disease without fever | 6 | 4.2 |
| NCD-Unspecifiable/other | 2 | 1.4 |
| NCD-Cardiovascular disorder (unspec/oth | 2 | 1.4 |
| NCD-Hypertension | 2 | 1.4 |
| NCD-Congestive heart disease | 11 | 7.8 |
| NCD-Ischaemic heart disease | 1 | 0.7 |
| NCD-Respiratory disorder (unspec/other) | 2 | 1.4 |
| NCD-Gastro intestinal disorder (unspec/ | 23 | 16.2 |
| NCD-Peptic ulcer disease | 3 | 2.1 |
| NCD-Liver cirrhosis | 16 | 11.3 |
| NCD-Acute abdomen including obstruction | 5 | 3.5 |
| NCD-Neoplasm (unspec/other) | 13 | 9.2 |
| NCD-Neoplasm - cervix/uterus | 4 | 2.8 |
| NCD-Neoplasm - liver | 5 | 3.5 |
| NCD-Neoplasm - GI exc liver | 9 | 6.3 |
| NCD-Neoplasm - lung | 1 | 0.7 |
| NCD-Genito urinary disorders (unspec/ot | 3 | 2.1 |
| NCD-Kidney disorder | 1 | 0.7 |
| NCD-Anaemia (unspec/other) | 1 | 0.7 |
| **Total** | **142** | **100.0** |

Supplementary Table 13: Percentage of assigned cause of deaths that were indeterminate by age group, sex and type of VA.

|  | Percentage indeterminate | | | | |
| --- | --- | --- | --- | --- | --- |
| Age group | Men | |  | Women | |
|  | InSilicoVA | Physician |  | InSilicoVA | Physician |
|  | n=28 | n=100 |  | n=29 | n=108 |
| 15-29 | 2.1 | 4.2 |  | 1.3 | 6.9 |
| 30-49 | 3.1 | 7.7 |  | 1.5 | 3.0 |
| 50-59 | 3.4 | 6.9 |  | 2.7 | 3.6 |
| 60-79 | 2.4 | 7.5 |  | 2.6 | 9.9 |
| 80+ | 0.0 | 15.9 |  | 4.2 | 20.3 |
| Total | 2.4 | 8.4 |  | 2.4 | 9.1 |

Supplementary Table 14: Leading cause of death by age group and type of VA assessment

| **Age group** |  | **MEN - Harmonised** |  |  |  |  |  |
| --- | --- | --- | --- | --- | --- | --- | --- |
|  |  | **InSilicoVA** |  |  | **Physician** |  |  |
|  |  |  | **n** | **%** | **Cause** | **n** | **%** |
| 50-59 (n=146) | 1 | TB/HIV/AIDS | 40 | 29% | TB/HIV/AIDS | 52 | 37% |
|  | 2 | Digestive neoplasms | 17 | 12% | Unspecified cardiac dis/Stroke | 15 | 11% |
|  | 3 | Acute resp infect incl pneumonia | 17 | 12% | Other and unspecified NCD | 10 | 7% |
|  | 4 | Other and unspecified infect dis | 12 | 9% | Acute abdomen | 8 | 6% |
|  | 5 | Unspecified cardiac dis/Stroke | 11 | 8% | Accid poisoning & noxious subs | 7 | 5% |
|  |  |  |  |  |  |  |  |
| 60-79 (n=293) | 1 | Unspecified cardiac dis/Stroke | 60 | 22% | Unspecified cardiac dis/Stroke | 76 | 27% |
|  | 2 | Digestive neoplasms | 44 | 16% | TB/HIV/AIDS | 37 | 13% |
|  | 3 | Other and unspecified infect dis | 38 | 14% | Other and unspecified NCD | 35 | 13% |
|  | 4 | Acute abdomen | 25 | 9% | Diabetes mellitus | 20 | 7% |
|  | 5 | Acute resp infect incl pneumonia | 22 | 8% | Other and unspecified neoplasms | 16 | 6% |
|  |  |  |  |  |  |  |  |
| 80+ (n=189) | 1 | Unspecified cardiac dis/Stroke | 44 | 24% | Unspecified cardiac dis/Stroke | 53 | 29% |
|  | 2 | Digestive neoplasms | 27 | 15% | Other and unspecified NCD | 24 | 13% |
|  | 3 | Acute resp infect incl pneumonia | 19 | 11% | Indeterminate | 21 | 12% |
|  | 4 | Respiratory neoplasms | 13 | 7% | Other and unspecified neoplasms | 9 | 5% |
|  | 5 | Other and unspecified infect dis | 12 | 7% | Acute resp infect incl pneumonia | 9 | 5% |

| **Age group** |  | **WOMEN - Harmonised** |  |  |  |  |  |
| --- | --- | --- | --- | --- | --- | --- | --- |
|  |  | **InSilicoVA** |  |  | **Physician** |  |  |
|  |  | **Cause** | **n** | **%** | **Cause** | **n** | **%** |
| 50-59 (n=112) | 1 | TB/HIV/AIDS | 35 | 32% | TB/HIV/AIDS | 43 | 39% |
|  | 2 | Digestive neoplasms | 15 | 14% | Unspecified cardiac dis/Stroke | 16 | 14% |
|  | 3 | Unspecified cardiac dis/Stroke | 12 | 11% | Other and unspecified NCD | 9 | 8% |
|  | 4 | Other and unspecified infect dis | 12 | 11% | Reproductive neoplasms MF | 7 | 6% |
|  | 5 | Reproductive neoplasms MF | 6 | 5% | Diabetes mellitus | 5 | 5% |
|  |  |  |  |  |  |  |  |
| 60-79 (n=352) | 1 | Unspecified cardiac dis/Stroke | 111 | 33% | Unspecified cardiac dis/Stroke | 128 | 38% |
|  | 2 | Digestive neoplasms | 32 | 9% | Other and unspecified NCD | 29 | 9% |
|  | 3 | Acute resp infect incl pneumonia | 30 | 9% | Reproductive neoplasms MF | 28 | 8% |
|  | 4 | Other and unspecified infect dis | 28 | 8% | TB/HIV/AIDS | 28 | 8% |
|  | 5 | Reproductive neoplasms MF | 25 | 7% | Other and unspecified infect dis | 24 | 7% |
|  |  |  |  |  |  |  |  |
| 80+ (n=237) | 1 | Unspecified cardiac dis/Stroke | 75 | 33% | Unspecified cardiac dis/Stroke | 91 | 40% |
|  | 2 | Other and unspecified infect dis | 34 | 15% | Indeterminate | 40 | 17% |
|  | 3 | Acute resp infect incl pneumonia | 21 | 9% | Other and unspecified NCD | 24 | 10% |
|  | 4 | Other and unspecified NCD | 17 | 7% | Acute resp infect incl pneumonia | 15 | 7% |
|  | 5 | Acute abdomen | 14 | 6% | Other and unspecified infect dis | 13 | 6% |

| **Age group** |  | **MEN** |  |  |  |  |  |
| --- | --- | --- | --- | --- | --- | --- | --- |
|  | **InSilicoVA** | |  |  | **Physician** |  |  |
|  | **Cause** | | **n** | **%** | **Cause** | **n** | **%** |
| 50-59 (n=146) | 1 | HIV/AIDS related death | 33 | 22% | CD-AIDS | 49 | 33% |
|  | 2 | Digestive neoplasms | 18 | 12% | NCD-Acute abdomen including obstruction | 8 | 5% |
|  | 3 | Acute resp infect incl pneumonia | 17 | 11% | Specific information missing on VAQ | 7 | 5% |
|  | 4 | Other and unspecified infect dis | 15 | 10% | EXT-Poisoning/exposure to noxious substance | 7 | 5% |
|  | 5 | Acute abdomen | 10 | 7% | CD-Pneumonia | 6 | 4% |
|  |  |  |  |  |  |  |  |
| 60-79 (n=293) | 1 | Digestive neoplasms | 45 | 15% | NCD-Congestive heart disease | 27 | 9% |
|  | 2 | Other and unspecified infect dis | 40 | 13% | CD-AIDS | 27 | 9% |
|  | 3 | Other and unspecified cardiac dis | 36 | 12% | NCD-Hypertension | 24 | 8% |
|  | 4 | Stroke | 29 | 10% | NCD-Cerebro vascular disease | 20 | 7% |
|  | 5 | Acute abdomen | 28 | 9% | NCD-Diabetes | 19 | 6% |
|  |  |  |  |  |  |  |  |
| 80+ (n=189) | 1 | Digestive neoplasms | 28 | 15% | NCD-Cerebro vascular disease | 21 | 11% |
|  | 2 | Other and unspecified cardiac dis | 24 | 13% | No significant pathology that would explain the death | 16 | 8% |
|  | 3 | Acute resp infect incl pneumonia | 19 | 10% | NCD-Congestive heart disease | 16 | 8% |
|  | 4 | Stroke | 18 | 9% | NCD-Gastro intestinal disorder (unspec/other) | 13 | 7% |
|  | 5 | Respiratory neoplasms | 15 | 8% | NCD-Hypertension | 11 | 6% |

| **Age group** |  | **WOMEN** |  |  |  |  |  |
| --- | --- | --- | --- | --- | --- | --- | --- |
|  | **InSilicoVA** | |  |  | **Physician** |  |  |
|  | **Cause** | | **n** | **%** | **Cause** | **n** | **%** |
| 50-59 (n=112) | 1 | HIV/AIDS related death | 33 | 29% | CD-AIDS | 39 | 35% |
|  | 2 | Digestive neoplasms | 15 | 13% | NCD-Neoplasm - cervix/uterus | 7 | 6% |
|  | 3 | Other and unspecified infect dis | 12 | 11% | NCD-Congestive heart disease | 6 | 5% |
|  | 4 | Stroke | 6 | 5% | NCD-Diabetes | 5 | 4% |
|  | 5 | Reproductive neoplasms MF | 6 | 5% | NCD-Hypertension | 5 | 4% |
|  |  |  |  |  |  |  |  |
| 60-79 (n=352) | 1 | Other and unspecified cardiac dis | 57 | 16% | NCD-Hypertension | 47 | 13% |
|  | 2 | Stroke | 52 | 15% | NCD-Congestive heart disease | 37 | 11% |
|  | 3 | Digestive neoplasms | 34 | 9% | NCD-Cerebro vascular disease | 32 | 9% |
|  | 4 | Other and unspecified infect dis | 31 | 9% | NCD-Neoplasm - cervix/uterus | 28 | 8% |
|  | 5 | Acute resp infect incl pneumonia | 31 | 9% | CD-AIDS | 16 | 5% |
|  |  |  |  |  |  |  |  |
| 80+ (n=237) | 1 | Other and unspecified cardiac dis | 48 | 20% | Indeterminate | 35 | 15% |
|  | 2 | Other and unspecified infect dis | 35 | 14% | NCD-Congestive heart disease | 32 | 14% |
|  | 3 | Stroke | 28 | 12% | NCD-Cerebro vascular disease | 31 | 13% |
|  | 4 | Acute resp infect incl pneumonia | 24 | 10% | NCD-Hypertension | 22 | 9% |
|  | 5 | Other and unspecified NCD | 17 | 7% | CD-Pneumonia | 15 | 6% |

Supplementary Panel 1: Case study 1 and 2 from a randomly selected set of case studies where InSilicoVA assigned the cause of death at digestive neoplasm and physician review did not.

| CASE 1: 56 years old, female | |  |
| --- | --- | --- |
| InSilicoVA CoD assignment [top two causes, with probability] | **1.Digestive neoplasms** (Probability: 0.9997879)  2. Acute abdomen (Probability: 0.000208) | |
| Physician assigned COD [final CoD, individual physician CoD and CoD harmonised to facilitate comparison with InSilicoVA] | Only underlying CoD assigned; no direct or contributory.  Underlying CoD:  **NCD- Neoplasm – cervix/uterus**  Physician 1= NCD- Neoplasm – cervix/uterus  Physician 2= NCD- Neoplasm – cervix/uterus  Physician 3= NCD**-** Neoplasm – cervix/uterus  *[Harmonised - Reproductive neoplasms MF]* | |
| Reported signs and symptoms from VA questionnaire | Physician 1: “cancer of cervix or uterus”. Physician 2: “cervical or uterine cancer”. Physician 3: “advanced cancer of the cervix”  Reports abdominal problems including severe lower abdominal pain and abdominal mass, information about vomiting is inconclusive (potentially 14 days of vomiting), however the deceased was reported to have 4 months of non-bloody diarrhoea and difficulty drinking for 14 days  The deceased was not reported to have menstrual bleeding  No respiratory symptoms or chest pain  No urinary symptoms  Systemically, she was reported to have weight loss and anaemia, severe on/off fever for 2 months  but no sweating  Past medical history included hypertension and previous surgery  She died in hospital | |
| Open narrative | The deceased had been in poor health four months prior to her death. She had been experiencing severe abdominal pains. She went to hospital where she underwent an operation to remove an abdominal tumour. Immediately after the operation she developed a vesico-vaginal-colonic fistula (passing stool mixed with urine and urine mixed with stool). Another operation was conducted but symptoms did not improve. | |
| CASE 2: 85 years old, male | | |
| InSilicoVA CoD assignment [top two causes, with probability] | **1. Digestive neoplasms** (Probability: 0.9999813)  2. Liver cirrhosis (Probability: 7.31E-06) | |
| Physician assigned COD [final CoD, individual physician CoD and CoD harmonised to facilitate comparison with InSilicoVA] | Underlying and direct CoD assigned; no contributory.  Underlying CoD:  **NCD - Gastro intestinal disorder** **(unspec/other)**  Physician 1= NCD- Gastro intestinal disorder (unspec/other)  Physician 2= NCD- Gastro intestinal disorder (unspec/other)  Physician 3= NCD**-** Gastro intestinal disorder (unspec/other)  *[Harmonised - Other and unspecified NCD]*  Direct CoD:  Physician 1= NCD- Gastro intestinal disorder (unspec/other)  Physician 2= NCD- Anaemia (caused by chronic CD)  Physician 3= NCD**-** Gastro intestinal disorder (unspec/other) | |
| Reported signs and symptoms from VA questionnaire | Physician 1: “upper GIT [Gastro Intestinal Tract] bleeding” “chronic liver disease”.  Physician 2: “anaemia” “tropical splenomegaly syndrome”.  Physician 3: “oesophageal varices (upper GI bleed)” “portal hypertension (with hypersplenism)”  The deceased was reported to have abdominal symptoms, predominantly 3 months of lower abdominal pain, 3 days of vomiting with blood, and possible bloody diarrhoea, but no abdominal swelling  They had chest pain but no cough or breathlessness. They were also reported to have ankle swelling and excessive urination  No fever was reported but the decease did experience weight loss and anaemia. They were also in a coma, but this was not reported to be a sudden onset  No past medical history was reported  The deceased went to hospital for treatment but died at home | |
| Open narrative | The deceased was in poor health for three months prior to their date of death. He had four episodes of vomiting blood, passing clotted blood, abdominal pains and leg ache for three months. He was seen in the hospital where he was diagnosed with tropical splenomegaly syndrome. He received at blood transfusion in hospital, however his condition did not improve, and he passed away. | |

Supplementary Panel 2: Case study 3-6 from a randomly selected set of case studies where InSilicoVA assigned the cause of death at digestive neoplasm and physician review did not.

| CASE 3: | |  |
| --- | --- | --- |
| InSilicoVA CoD assignment [top two causes, with probability] | **1. Digestive neoplasms** [P: 0.9999655]  2. Reproductive neoplasms MF [P: 0.0000344] | |
| Physician assigned COD (final CoD, individual physician CoD and CoD harmonised to facilitate comparison with InSilicoVA) | Underlying and direct CoD assigned (although direct CoD only provided by one physician); no contributory.  Underlying CoD:  **NCD - Neoplasms (unspec/other)**  Physician 1= NCD- Neoplasms (unspec/other)  Physician 2= NCD- Gastro intestinal disorder (unspec/other)  Physician 3= NCD- Neoplasms (unspec/other)  *[Harmonised - Other and unspecified neoplasms*]  Direct CoD:  Physician 1= NCD- Anaemia (unspec/other) | |
| Reported signs and symptoms | Physician 1: “Unspecific Cancer with Distant Metastases”  Physician 2: “anaemia unspecified” and “GI unspecified”  Physician 3: “metastatic cancer”  This woman was reported to have been chronically ill for around 10 years. She had weight loss, wasting and anaemia which likely lead her to having a blood transfusion at some point  A month prior to her death, she experienced abdominal swelling, severe generalised abdominal pain and was noted to have an abdominal mass. She was also reported to have skin ulcers of unknown duration  In the final days, she experienced 3 days of increased difficulty breathing including rapid breathing and shortness of breath on exertion and when lying flat  She also experienced a severe on/off fever for 2 days with diarrhoea and vomiting, some of which contained blood  She had difficulty drinking and developed a headache and sudden coma  She did not experience a cough, chest pain, signs of meningitis, jaundice, urinary retention, haematuria or PV bleeding. She did not show signs of peripheral oedema. Besides the abdominal mass, there were no other reported lumps  Regarding social history, she did not smoke or drink excessively. She was previously diagnosed with heart disease and cancer. She did not have HIV or TB  The deceased was taken to hospital via motorised transport. A mobile phone was used to call for help however the family found the costs of hospitalisation very high  The history was given by a close relative. She died in hospital | |
| Open narrative | [name] had a chronic cancer ulcer at the back which used to bleed. She had the condition for the past 10 years and was on home-based care. Terminally she developed abdominal pain and swelling and had palpitation for one month. Abdominal tap was done at Mzuzu central hospital blood a stained the fluid got aspirated. Later she also developed vomiting blood fresh plus clotted, fever, headache and difficulties in breathing and passing bloody stools for two days. God transfused at KDH but later passed away. | |
| General comments | Both physician review and InSilicoVA agreed that the underlying cause of death in this person was cancer involving the abdomen, however InSilicoVA has given a much more specific diagnosis. Given that comments left by physicians suggest there may have been metastatic spread of the cancer, it suggests InSilicoVA may have struggled to distinguishing primary abdominal neoplasm from metastatic spread. | |
| CASE 4: | | |
| InSilicoVA CoD assignment [top two causes, with probability] | **1. Digestive neoplasms [**P: 0.9999285]  2. Liver cirrhosis [P: 0.0000712] | |
| Physician assigned COD [final CoD, individual physician CoD and CoD harmonised to facilitate comparison with InSilicoVA] | Underlying and direct CoD assigned (although direct CoD only provided by one physician); no contributory.  Underlying CoD:  **NCD - Liver cirrhosis**  Physician 1= NCD- Gastro intestinal disorder (unspec/other)  Physician 2= NCD- Liver cirrhosis  Physician 3= NCD- Liver cirrhosis  *[Harmonised -* Liver cirrhosis]  Direct CoD:  Physician 3= NCD- Gastro intestinal disorder (unspec/other) | |
| Reported signs and symptoms | Physician 1: “Chronic liver disease? Chronic hepatitis”  Physician 2: “distended abdomen, jaundice, vomiting blood USS suggestive of cirrhosis”  Physician 3: “Oesophageal varices (upper [gastrointestinal tract] bleeding)” “liver cirrhosis”  This man was reported to have a mild, fluctuating fever for 14 days, with 3 days of difficulty breathing. These symptoms were on the background of 12-months of reported abdominal swelling, lower abdominal pain, and an abdominal mass.  He was reported to be jaundice with associated ankle swelling and excessive urination. He was otherwise not reported to have diarrhoea or vomiting despite the latter being noted in the physician comments  There were no reports of cardiac, respiratory or neurological symptoms including no change in mental state. He was not reported to have any lumps, skin changes or skin ulcers  He was not reported to have any weight loss or anaemia. He was also not reported to have any past medical history including HIV, diabetes, hypertension, or any previous surgeries.  This man was taken to the hospital where he passed away  These responses were given by a close family member | |
| Open Narrative | Deceased had been in poor health since 1995 when he developed vomiting blood and abdominal distension. He had been receiving treatment from various health facilities. In 01/2013, he was seen at KDH where an abdominal scan was done, was told had damaged liver. In December 2013, he got admitted at KDH after he also developed the yellowish discolouration of eyes and severe abdominal distension. He died after a week admission. | |
| General Comments | There is some discrepancy between physician assigned cause of death. All three physicians and InSilicoVA agree the cause of death is associated with an abdominal pathology, however InSilicoVA suggests a cancer was involved whereas physician review does not suggest this. As noted in one of the physician comments and the open narrative, this patient appears to have undergone further investigations for their disease. The findings of their scan may have been available to the physicians but has not been captured by the VA questionnaire | |
| CASE 5: | | |
| InSilicoVA CoD assignment [top two causes, with probability] | **1. Digestive neoplasms [**P: 0.6243493]  2. Reproductive neoplasms MF [P: 0.3711926] | |
| Physician assigned COD [final CoD, individual physician CoD and CoD harmonised to facilitate comparison with InSilicoVA] | Underlying and contributory CoD assigned (although contributory CoD only provided by one physician); no direct.  Underlying CoD:  **NCD- Neoplasms (unspecified/ other)**  Physician 1= NCD- Genito urinary disorders (unspec/other)  Physician 2= NCD- Neoplasm (unspec/other)  Physician 3= NCD- Neoplasm (unspec/other)  *[Harmonised - Other and unspecified neoplasms]*  Direct CoD:  Physician 1= NCD- Genito urinary disorders (unspec/other) | |
| Reported signs and symptoms | Physician 1: “Abnormal Vaginal Discharge” “lower abdominal pain”  Physician 2: “[seen] with wasting and lack of response to antibiotics that likely gynae cancer”  Physician 3: “gynaecological cancer - vaginal discharge, abdominal pain, peripheral oedema, wasting”  This woman experienced 2 days of vomiting and 14 days of difficulty drinking. She was reported to have 6 months of severe lower abdominal pain, but there was no abdominal mass or swelling.  She did have vaginal discharge, but this was not bloody, and she did not have post-menopausal bleeding.  In her final illness, she was not reported to have a fever or any cardiac, respiratory, or neurological symptoms.  Systemically she was reported to have weight loss and muscle wasting with night sweats. She experienced ankle swelling and potentially some skin ulcers  She was not reported to have any past medical history however she underwent HIV testing and was referred for anti-retroviral therapy in 2017, the same year she passed away.  The deceased was taken to the hospital for treatment but died at home  Response given by relatives | |
| Open narrative | The woman died at the age of 72 years. She was well until 6/12 prior to the date of death when she developed pussy virginal discharge 6/12, lower abdominal pains 6/12, painful urination 6/12, and swelling of feet 6/12. Had visited various health facilities and was put on several antibiotics to no improvement. She was advised to be on home-based care. Terminally she was bed ridden and had developed pressure sores on the left buttocks. Died at home on 09/10/2017 | |
| General comments | As with previous cases, InsilicoVA has given a more specific cause of death than physician review. Furthermore, the open narrative gives a more nuance description of her symptoms such as a long history of pus-filled vaginal discharge which can help physicians understand that there may have been an underlying gynaecological malignancy causing a chronic infection | |
| CASE 6: | | |
| InSilicoVA CoD assignment [top two causes, with probability] | **1. Digestive neoplasms [**P: 0.9847353**]**  2. Other and unspecified neoplasms **[**P: 0.0128662] | |
| Physician assigned COD [final CoD, individual physician CoD and CoD harmonised to facilitate comparison with InSilicoVA] | Only underlying CoD assigned; no direct or contributory.  Underlying CoD:  **NCD- Neoplasm - liver**  Physician 1= NCD- Liver cirrhosis  Physician 2= NCD- Neoplasm - liver  Physician 3= NCD- Neoplasm - liver  *[Harmonised -* *Other and unspecified neoplasms]* | |
| Reported signs and symptoms | Physician 1: “liver cirrhosis”  Physician 2: “rapid progression”  Physician 3: “Hepatoma or liver malignancy?”  This man was reported to have a headache and 7 days of neck stiffness prior to passing away. They had experienced abdominal swelling for 25 days with associated jaundice and ankle swelling. They did not have any abdominal masses, pain, diarrhoea or vomiting  They did not have any cardiac, respiratory, or other neurological symptoms  The man experienced weight loss and wasting but no anaemia. They had a month of difficulty drinking. They had no significant past medical history.  The man was taken to hospital however, they died at home. The VA was reported by a close family member | |
| Open narrative | The deceased was previously well and you two months or so before he died, he lost his appetite for a week then had diarrhoea and vomiting for a few days these subsided, then he gradually started having his abdomen swell. By this time he had developed yellowish appearance of eyes. Yellowish appearance continued until he was taken to CRH for help after getting treatment there he was told to go to KDH the following day, at KDH he was admitted for one week and requested discharge this was granted. By this time he was better after two weeks at home his neck got stiffness and rigid and also had moderate to severe headache for one week he did not go to the hospital again until he died at home. | |
| General comments | From the VA responses and open narrative, the symptoms appear to be in keeping with a liver pathology with associated liver failure (jaundice with central and peripheral oedema). Physician review and InSilicoVA cause of death assignment are very similar. | |
